# Cold exposure aggravates vein occlusion through non-shivering thermogenesis-induced thrombocytopoiesis

**DOI:** 10.1038/s41422-026-01280-2

**Published:** 2026-07-30

**Authors:** Sisi Xie, Kaihong Chen, Xiaoting Sun, Ying Ye, Ruibo Chen, Chenyu Jiang, Mingjia Chen, Xue Lv, Huilan Li, Ying Liao, Weihua Chen, Lin Deng, Linli Cai, Yu Liang, Qiaoling Wei, Ji Zuo, Guohua Yu, Libo Yang, Jiansong Ji, Li Lin, Wei Tao, Chen Zhao, Yunlong Yang, Yihai Cao

**Affiliations:** 1 Department of Cardiology, Basic Scientific Research Center, Longyan First Hospital Affiliated to Fujian Medical University, Longyan, Fujian, China; 2 School of Pharmaceutical Science, Wenzhou Medical University, Wenzhou, Zhejiang, China; 3 Department of Oral Implantology, Stomatological Hospital and Dental School of Tongji University, Shanghai Engineering Research Center of Tooth Restoration and Regeneration, Shanghai, China; 4 Department of Cellular and Genetic Medicine, School of Basic Medical Sciences, Fudan University, Shanghai, China; 5 State Key Laboratory of Experimental Hematology, National Clinical Research Center for Blood Diseases, Institute of Hematology & Blood Diseases Hospital, Chinese Academy of Medical Sciences & Peking Union Medical College, Tianjin, China; 6 Eye Institute and Department of Ophthalmology, Eye & ENT Hospital; Key Laboratory of Myopia and Related Eye Diseases, NHC; Key Laboratory of Myopia and Related Eye Diseases, Chinese Academy of Medical Sciences; Shanghai Key Laboratory of Visual Impairment and Restoration, Fudan University, Shanghai, China; 7 Wei Fang People’s Hospital, Kui Wen District, Weifang, Shandong, China; 8 The Affiliated Taian City Central Hospital of Qingdao University, Taian, Shandong, China; 9 Key Laboratory of Imaging Diagnosis and Minimally Invasive Intervention Research, Institute of Imaging Diagnosis and Minimally Invasive Intervention Research, the Fifth Affiliated Hospital of Wenzhou Medical University, Lishui, Zhejiang, China; 10 Center for Nanomedicine and Department of Anesthesiology, Brigham and Women’s Hospital, Harvard Medical School, Boston, MA USA; 11 Department of Microbiology, Tumor and Cell Biology, Karolinska Institutet, Solna, Sweden

**Keywords:** Mechanisms of disease, Cell biology

## Abstract

Vein occlusion (VO), including deep venous thrombosis (DVT) and retinal vein occlusion (RVO), is a common cause of multiple diseases that severely compromise the quality of life of affected individuals. Epidemiological evidence indicates that VO prevalence increases in cold seasons, yet the underlying mechanism remains unknown. Here, we show that cold exposure markedly elevates peripheral platelet counts, thereby aggravating VO in mouse models. Cold-augmented thrombocytopoiesis depends on the activation of adipose thermogenesis and subsequent increase in circulating free fatty acid (FFA) levels. Mechanistically, FFA-β-oxidation promotes acetyl-CoA production, which upregulates and stabilizes C/EBPα by shifting the balance between p300 acetyltransferase and SIRT1 deacetylase. Acetyl-C/EBPα transcriptionally upregulates GATA-1 and NF-E2 for megakaryocyte maturation and platelet production. Depletion of adipose triglyceride lipase PNPLA2, megakaryocyte-specific knockout of key β-oxidation enzyme CPT1α, or pharmacological inhibition of CPT1α and p300 abolishes cold-augmented thrombocytopoiesis and alleviates DVT and RVO in mouse models. In healthy volunteers, tolerable cold exposure activates adipose thermogenesis, increases circulating FFA levels, and increases platelet counts. Moreover, a retrospective cohort study of 425 patients reveals elevated platelet counts and higher DVT incidence during cold seasons. Similarly, increased platelet counts are observed in 448 patients with RVO at the time of diagnosis in cold seasons. Our study provides novel mechanistic insights into the increased VO risk induced by cold exposure and proposes a new therapeutic paradigm for VO by targeting megakaryocyte metabolism.

## Introduction

Deep vein thrombosis (DVT) and its life-threatening complication, pulmonary embolism, affect nearly 10 million people annually^1^ and account for substantial mortality and disability, thus imposing a heavy global disease burden.^2^ Vein occlusion (VO), which includes DVT, is the third most common vascular disease and commonly occurs in the large veins of the legs. Small VO can also cause severe pathological consequences. For example, retinal vein occlusion (RVO), a major cause of retinal vascular abnormality and visual loss in young adults, affects more than 16 million people worldwide.^3^ VO can be triggered by diverse pathological processes such as trauma, surgery, immobility, and various diseases, including cancer. Platelets play a central role in VO development, alongside endothelial dysfunction, inflammation, and coagulation.^2^ Platelet activation by agonists such as thrombin, adenosine diphosphate (ADP), and collagen drives platelet aggregation and ultimately leads to VO. Interestingly, since the 1980s, accumulating evidence has established a strong causative link between increased DVT incidence and cold seasons, with a peak during winter.^4–7^ Similar correlations have been documented in RVO.^8–10^ Numerous mechanisms have been proposed to explain this phenomenon, including cold-induced increases in systolic blood pressure,^11^ blood viscosity,^12^ and coagulation factors,^13^ as well as reduced vitamin D levels due to insufficient sunlight exposure during winter.^14^ However, these mechanisms remain speculative and lack robust supporting evidence. To better understand the seasonal pattern of VO, detailed characterization of molecular signaling pathways and their functional validation are warranted.

In mammals, cold exposure induces adaptive skeletal muscle shivering thermogenesis and adipose non-shivering thermogenesis (NST). NST is executed by a conserved mitochondrial uncoupling protein 1 (UCP1)-dependent mechanism in browning white adipose tissue (WAT) and activated brown adipose tissue (BAT).^15–17^ Thermogenic adipose tissues are considered a sink for excessive metabolites, thereby exerting beneficial effects in alleviating adiposity and insulin resistance. During NST, the balance of lipolysis of triacylglycerol (TG) and re-esterification of free fatty acids (FFAs) is disrupted in adipose tissue, triggering a dynamic shift toward catabolism. Circulating FFAs can be utilized via UCP1-mediated proton leak respiration.^18,19^ However, the involvement of FFAs in non-thermogenic processes under cold exposure has been largely overlooked.

Megakaryocytes (MKs), as platelet precursor cells, represent a minor subpopulation of myeloid cells that primarily reside in bone marrow (BM) and peripheral blood (PB).^20^ MKs originate from hematopoietic stem cells (HSCs) that undergo sequential differentiation into burst-forming units and colony-forming units.^21^ Expansion of MK cytoplasmic and membrane content is a prerequisite for platelet biogenesis. Prior to platelet release, MKs enlarge and accumulate high levels of ribosomes and DNA to support platelet production.^22^ Additionally, formation of the demarcation membrane system (DMS), an interconnected internal membrane network of cisternae and tubules,^23^ enables the assembly of individual platelets and their subsequent release from mature MKs. MK maturation and platelet production are regulated by various extracellular stimuli, including thrombopoietin (TPO),^24^ angiogenic factors,^25^ estrogen,^26^ microRNAs,^27^ and BM extracellular matrix.^28^ In MKs, several transcription factors (TFs), including GATA-1, FOG-1, and NF-E2, have been identified as key drivers of thrombocytopoiesis.^29,30^ GATA-1 participates in both MK formation and proliferation. Specific deletion of the *Gata1* gene in MKs in mice results in thrombocytopenia, accompanied by accumulation of immature MKs in BM.^31^ Similarly, in humans, functional mutations of GATA-1 that impair its interaction with FOG-1 also cause thrombocytopenia.^32^ Genetic deletion of NF-E2 in mice is lethal due to severe hemorrhage caused by thrombocytopenia.^33^ Despite normal MK maturation, proplatelet formation is completely defective in NF-E2-null mice.^34^

Derived from HSCs, MKs are morphologically distinguished by their large size and polyploidy,^35^ which are consistent with the high, constant demand for platelet production. Studies from our group and others suggest that the MK metabolic program entails arachidonic acid metabolism,^36^ glucose metabolism,^37^ and ketone body metabolism.^38^ We hypothesized that sustained platelet biogenesis may require a high rate of metabolism in MKs. To the best of our knowledge, the effects of cold exposure and its metabolic impacts on MKs and platelets have not been investigated.

In this study, we discovered that cold exposure markedly increased platelet counts in PB via an adipose NST-dependent lipolysis mechanism. FFAs released by NST-induced lipolysis markedly increased the expression and acetylation of C/EBPα in MKs, which targets GATA1 and NF-E2 to promote platelet production. Conversely, blocking β-oxidation, lipolysis, or acetyltransferase activity completely ablated the cold-induced thrombocytopoiesis. Using DVT and RVO mouse models, we provided evidence that targeting the lipolysis-β-oxidation-acetylation axis effectively reduced cold-augmented VO. In human studies, we showed that tolerable cold exposure in healthy adult volunteers increased circulating FFAs and platelet counts. Importantly, elevated platelet counts were observed in DVT and RVO patients during cold seasons, which likely contributes to increased VO incidence. Together, these data provide mechanistic insights into cold exposure-aggravated VO and offer a previously unrecognized paradigm for cold-associated VO by targeting the lipolysis-β-oxidation-acetylation axis in MKs.

## Results

### Cold exposure increases peripheral platelet counts

**Fig. 1:**
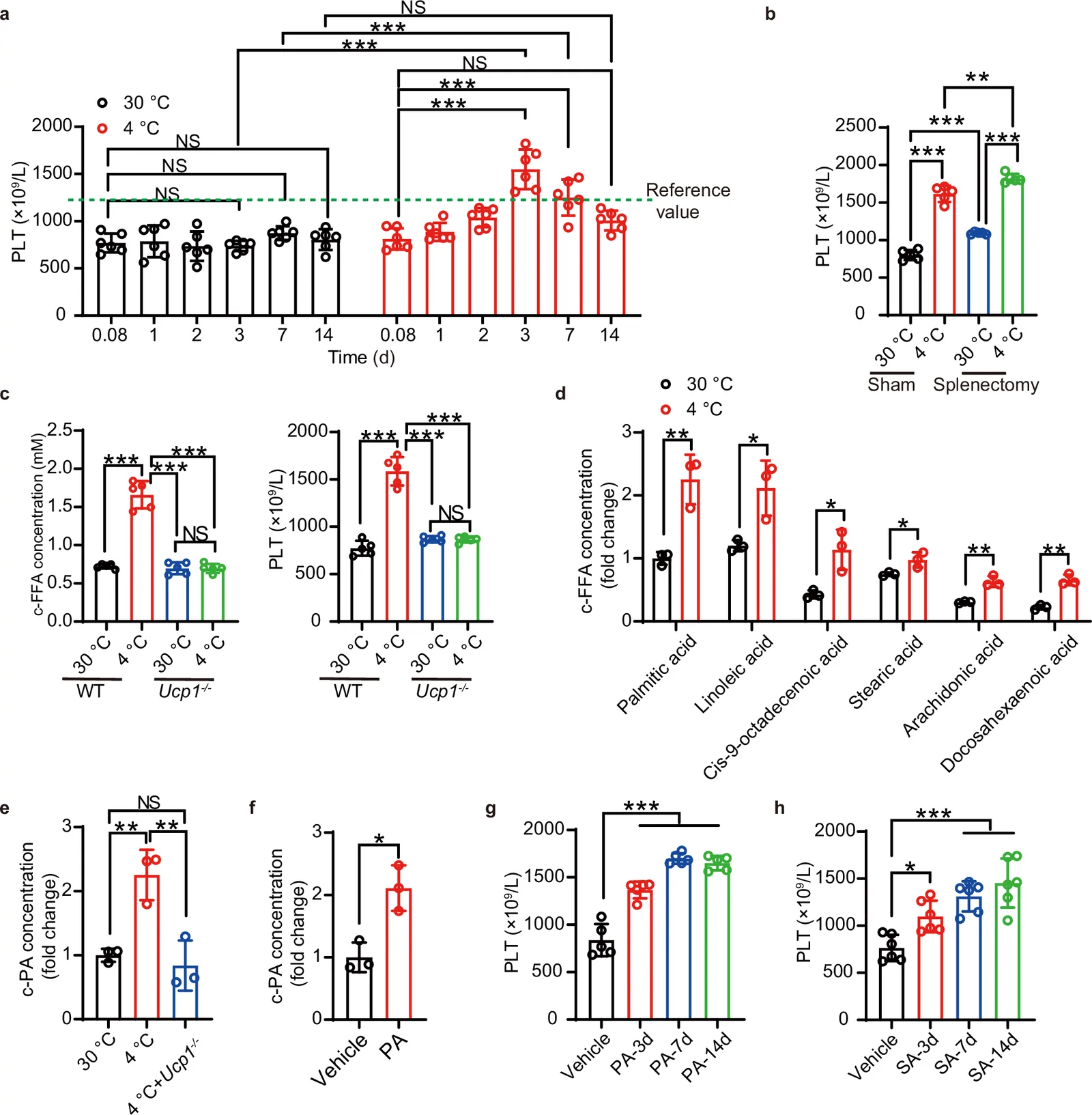
Cold exposure promotes platelet counts and c-FFA levels in mice. **a** Analysis of platelets (PLT) in PB from mice under 30 °C or 4 °C exposure at various time points (*n* = 6 mice per group). Day 3 was chosen for the following experiments. **b** Sham operation or splenectomy was performed, and mice were then exposed to 30 °C or 4 °C. Quantification of PLT in the indicated groups (*n* = 5 mice per group). **c** c-FFA levels and PLT counts in WT and *Ucp1*^*–/–*^ mice under 30 °C and 4 °C conditions (*n* = 5 mice per group). **d** Quantification of top 6 FFAs in serum of 30 °C- and 4 °C-exposed mice (*n* = 3 samples per group). **e** c-PA levels in 30 °C-exposed WT mice, 4 °C-exposed WT mice, and 4 °C-exposed *Ucp1*^*–/–*^ mice (*n* = 3 mice per group). c-PA levels in WT mice are the same as in **d**. **f** Quantification of c-PA in serum of mice administered vehicle or PA for 7 days (*n* = 3 mice per group). **g** Analysis of PLT in PB from mice administered vehicle or PA for 3, 7, or 14 days (*n* = 5 mice per group). **h** Analysis of PLT in PB from mice administered vehicle or SA for 3, 7, or 14 days (*n* = 6 mice per group). *P < 0.05; **P < 0.01; ***P < 0.001. NS not significant. Data are presented as mean ± SD.

Cold seasons are strongly associated with increased VO incidence, and platelets play a crucial role in thrombosis. To investigate the link between cold exposure and platelets in thrombosis, 10–13-week-old C57BL/6 mice were randomized and acclimated to either 30 °C for thermoneutrality or 4 °C for cold exposure. Interestingly, from day 3 of cold exposure, peripheral platelet counts increased to over 1200 × 10^9^/L, exceeding the upper limit of the mouse platelet reference range,^39^ compared to thermoneutral controls (Fig. 1a). Platelet distribution width (PDW) and mean platelet volume (MPV) were not altered (Supplementary information, Fig. S1a), and the reticulated platelet fraction was significantly increased (Supplementary information, Fig. S1b). Transient cold exposure did not increase platelet counts. Platelet counts peaked on day 3 and gradually declined during prolonged exposure, but they remained elevated after 1 week of cold exposure (Fig. 1a). These findings indicate that cold exposure induces thrombocytopoiesis in mice.

Electron microscopy of platelets revealed no alterations in morphology, size, or granular content (Supplementary information, Fig. S1c). Consistently, measurements of platelet activation, including clot retraction and platelet aggregometry using equal numbers of platelets, showed no significant differences between the cold-exposed and thermoneutral groups (Supplementary information, Fig. S1d, e).

It appeared that cold-induced thrombocytopoiesis was specifically restricted to platelets and that other blood parameters of red blood cells (RBCs) and white blood cells (WBCs) were nearly identical in the 30 °C and 4 °C groups (Supplementary information, Table S1). Parameters of the intrinsic, extrinsic, and common pathways of the coagulation cascade, including prothrombin time, activated partial thromboplastin time (aPTT), thrombin time, and fibrinogen content, remained unchanged (Supplementary information, Fig. S1f). Whole blood thromboelastography showed that coagulation factors and fibrinogen remained unchanged, whereas maximum amplitude was significantly increased, supporting a quantitative increase in platelets (Supplementary information, Fig. S1g). Since the spleen is the primary organ mediating platelet clearance, we analyzed platelets in splenectomized mice. Compared with the sham operation (control), splenectomy did not alter cold-induced increases in platelet counts (Fig. 1b). These findings suggest that platelet clearance is not involved in cold-induced thrombocytopoiesis.

### Adipose NST alters circulating FFAs and mediates thrombocytopoiesis

Cold exposure is known to promote browning of WAT, activate BAT, and trigger lipolysis. Consistent with this, exposing adult mice to 4 °C markedly induced WAT browning, activated BAT, and upregulated multiple browning markers, including Uncoupling protein 1 (*Ucp1*), Cytochrome c oxidase subunit 4 (*Cox4*), PR domain containing 16 (*Prdm16*), Peroxisome proliferator-activated receptor gamma (*Pparg*), and Cell death-inducing DFFA-like effector a (*Cidea*) (Supplementary information, Fig. S2a–c). To study the causative link between adipose NST and thrombocytopoiesis under cold exposure, *UCP1* knockout (*Ucp1*^*−/−*^*)* mice, which lack the ability to enhance adipose browning under cold exposure, were used (Supplementary information, Fig. S2d). Exposing wild-type (WT) mice to 4 °C for 3 days significantly increased circulating FFA (c-FFA) levels from 0.7 mM to ~2 mM (Fig. 1c). Genetic deletion of *Ucp1* completely normalized c-FFA levels to those of the 30 °C-exposed group (Fig. 1c). Surprisingly, consistent with the reduced c-FFA levels, *Ucp1* knockout also eliminated cold-induced increases in platelet counts, completely abolishing these cold-triggered effects (Fig. 1c). Consistently, β3-adrenergic receptor blockade completely abolished cold-induced *Ucp1* expression in thermogenic adipose tissues and cold-elevated platelet counts (Supplementary information, Fig. S2e, f). These results indicate a causal link between adipose NST and thrombocytopoiesis.

We next implemented a lipidomics approach to analyze changes in c-FFA composition during cold exposure. Under thermoneutrality, the main c-FFAs were linoleic acid, palmitic acid (PA), cis-9-octadecenoic acid, and stearic acid. Cold exposure significantly increased concentrations of most c-FFA species. Quantification of FFAs showed that PA was the dominant c-FFA under cold exposure, with levels more than doubling (Fig. 1d). As observed for total c-FFA levels, *Ucp1* deletion completely abrogated the cold-induced increase in c-PA (Fig. 1e). Furthermore, intraperitoneal administration of 1.2 mM/kg PA under thermoneutrality increased c-PA levels and induced thrombocytopoiesis to a degree comparable to cold exposure (Fig. 1f, g; Supplementary information, Table S1). Consistently, measurements of platelet activation showed no significant differences between vehicle- and PA-treated groups (Supplementary information, Fig. S3a, b). Stearic acid, another abundant c-FFA under cold exposure (Fig. 1d), exhibited effects similar to those of PA (Fig. 1h), supporting that c-FFAs mediate NST-induced thrombocytopoiesis. These findings demonstrate that NST-induced lipolysis alters c-FFA profiles and that FFAs, with PA as a major component, mediate cold-induced thrombocytopoiesis.

### MK β-oxidation-dependent platelet production

**Fig. 2:**
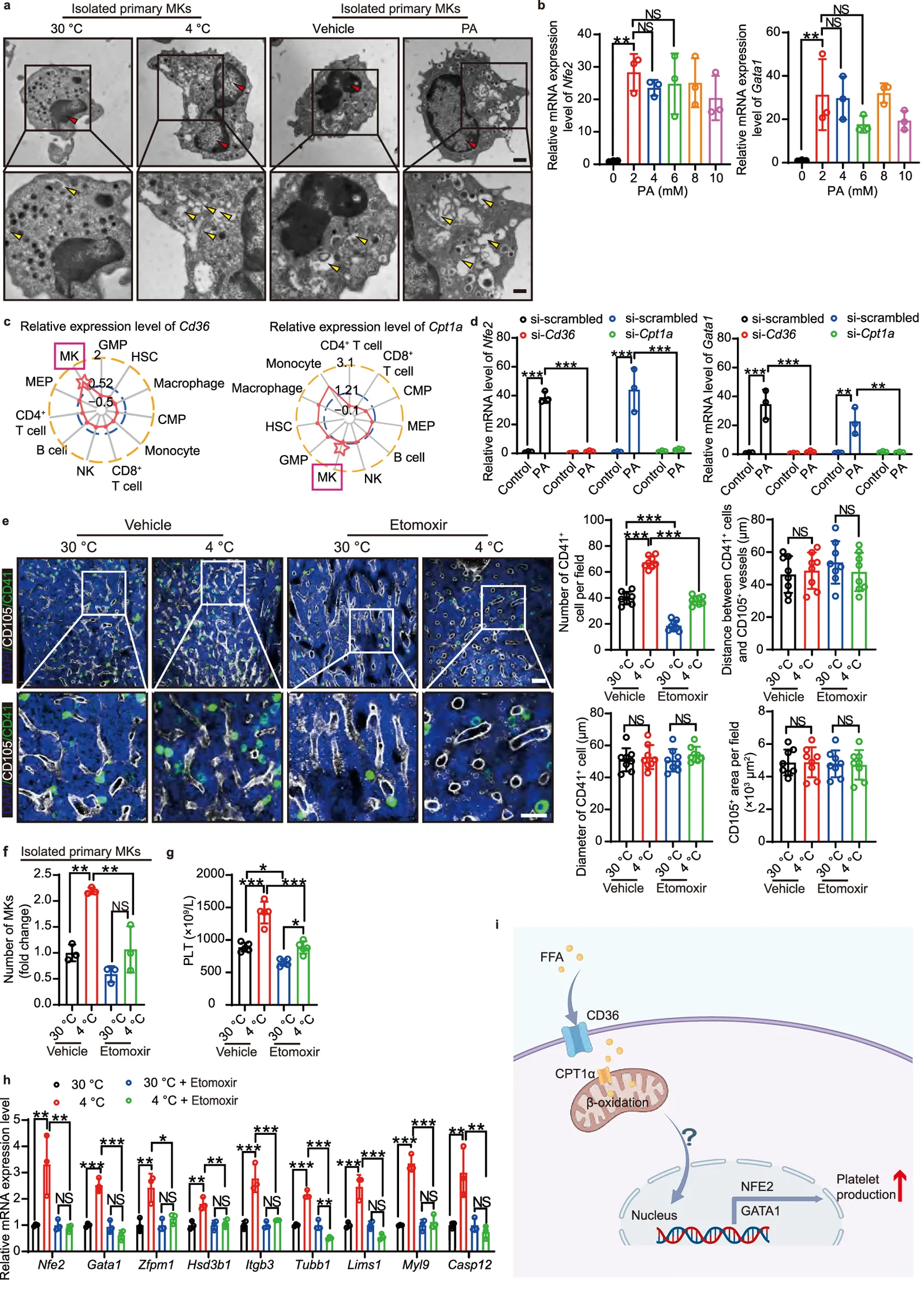
PA promotes thrombocytopoiesis via the CD36-CPT1α axis. **a** Representative transmission electron microscopy micrographs of MKs from 30 °C- or 4 °C-exposed mice and vehicle- or PA-treated mice. Red arrowhead: nucleus. Yellow arrowheads: pre-DMS. Scale bar in upper panel, 1 μm. Scale bar in lower panel, 500 nm. **b** qPCR quantification of *Nfe2* and *Gata1* in primary MKs treated with various concentrations of PA (*n* = 3 samples per group). **c** mRNA expression levels of *Cd36* and *Cpt1a* among various cell types in the hematopoietic stem cell lineage. Data were collected and analyzed from the Atlas of Mouse Blood Cells. Red star indicates MKs. **d** qPCR quantification of *Nfe2* and *Gata1* in si-scrambled-, si-*Cd36*-, or si-*Cpt1a*-transfected primary MKs under vehicle or PA treatment (*n* = 3 samples per group). **e** Representative whole-mount micrographs of CD41 (green), CD105 (white), and DAPI (blue) in the BM of vehicle- or etomoxir-treated mice under 30 °C or 4 °C. Scale bar: 100 μm. Quantification of the number of CD41^+^ cells per field, the average diameter of CD41^+^ cells, the average distance between CD41^+^ cells and CD105^+^ vessels, and the CD105^+^ area per field (*n* = 8 random fields per group). **f** Quantification of isolated MKs in the BM of vehicle- or etomoxir-treated mice under 30 °C or 4 °C (*n* = 3 mice per group). **g** PLT count in PB from vehicle- or etomoxir-treated mice under 30 °C or 4 °C (*n* = 5 mice per group). **h** Thrombocytopoiesis-associated gene expression in isolated primary MKs from vehicle- or etomoxir-treated mice under 30 °C or 4 °C (*n* = 3 samples per group). **i** Schematic model of FFA-induced platelet production. Cold-induced c-FFAs are transported into MKs for β-oxidation via CD36 and CPT1α. CPT1α-mediated β-oxidation is required for cold-induced thrombocytopoiesis (Created in BioRender. Ruibo, C. (2026) https://BioRender.com/bojdcrx). *P < 0.05; **P < 0.01; ***P < 0.001. NS not significant. Data are presented as mean ± SD.

Primary MKs were isolated from 30 °C- or 4 °C-exposed mice using a density gradient centrifugation-based method (Supplementary information, Fig. S3c, d), or were stimulated with PA in vitro, to study the effects on MK maturation. Electron microscopy showed that MKs from cold-exposed mice and PA-stimulated primary MKs exhibited morphological features of mature MKs, including enlarged nuclei and DMS formation (Fig. 2a).

To analyze MK maturation at the molecular level, we profiled expression levels of TFs that are known to be associated with MK maturation. Interestingly, TF mRNA expression profiling of isolated MKs from BM showed that the key MK maturation TF GATA-1^40^ and the platelet production TF NF-E2^33^ were the most upregulated in cold-exposed mice (Supplementary information, Fig. S3e). Consistent with morphological maturation, *Nfe2* and *Gata1* were markedly increased in 2 mM PA-stimulated MKs (Fig. 2b). This PA concentration matched the cold-induced c-FFA levels in vivo (Fig. 1d). To explore the causative link between PA and TF upregulation, a siRNA knockdown approach was employed to downregulate the expression of PA metabolism-associated genes, which are highly expressed in MKs relative to other BM cells (Fig. 2c; Supplementary information, Fig. S3f). Knockdown of *Cd36*, the key FFA transporter, completely abolished PA-induced upregulation of *Nfe2* and *Gata1* (Fig. 2d). Similarly, si*Cpt1a*, targeting the mitochondrial β-oxidation pathway, also completely abolished the PA-induced effect (Fig. 2d). These data confirm that FFA uptake and β-oxidation are essential for transcriptional regulation of MK maturation and platelet production.

To study the impact of cold-induced increases in c-FFA levels on MKs in vivo, BM was analyzed in the presence or absence of a CPT1α inhibitor. Consistent with increased peripheral platelets, cold exposure substantially increased CD41^+^ MKs in BM (Fig. 2e), suggesting lipolysis-driven MK expansion. Indeed, inhibition of β-oxidation by etomoxir, a CPT1α-specific inhibitor, completely abrogated cold-induced increases in BM CD41^+^ MKs (Fig. 2e). Isolation of BM MKs validated cold-induced MK increases and etomoxir’s inhibitory effect (Fig. 2f). Consistent with the MK findings, etomoxir also mitigated cold-induced thrombocytopoiesis (Fig. 2g) without altering the inert states of the platelets (Supplementary information, Fig. S3g). Two methods of MK isolation consistently showed that cold exposure upregulated expression of multiple platelet production-related genes, including *Nfe2*, *Gata1*, zinc finger protein, FOGfamily member 1 (*Zfpm1*), hydroxy-delta-5-steroid dehydrogenase, 3 beta- and steroid delta-isomerase 1 (*Hsd3b1*), integrin subunit beta 3 (*Itgb3*), tubulin beta 1 class VI (*Tubb1*), LIM zinc finger domaincontaining 1 (*Lims1*), myosin light chain 9 (*Myl9*), and caspase 12 (*Casp12*) (Fig. 2h; Supplementary information, Fig. S3h). Etomoxir efficiently blocked the expression of these genes (Fig. 2h). Similarly, genetic deletion of *Ucp1* ablated the cold-induced upregulation of these genes (Supplementary information, Fig. S3i). Together, these findings demonstrate a β-oxidation-dependent mechanism underlying cold-induced thrombocytopoiesis (Fig. 2i).

### C/EBPα-dependent transcriptional regulation of platelet production-related genes

**Fig. 3:**
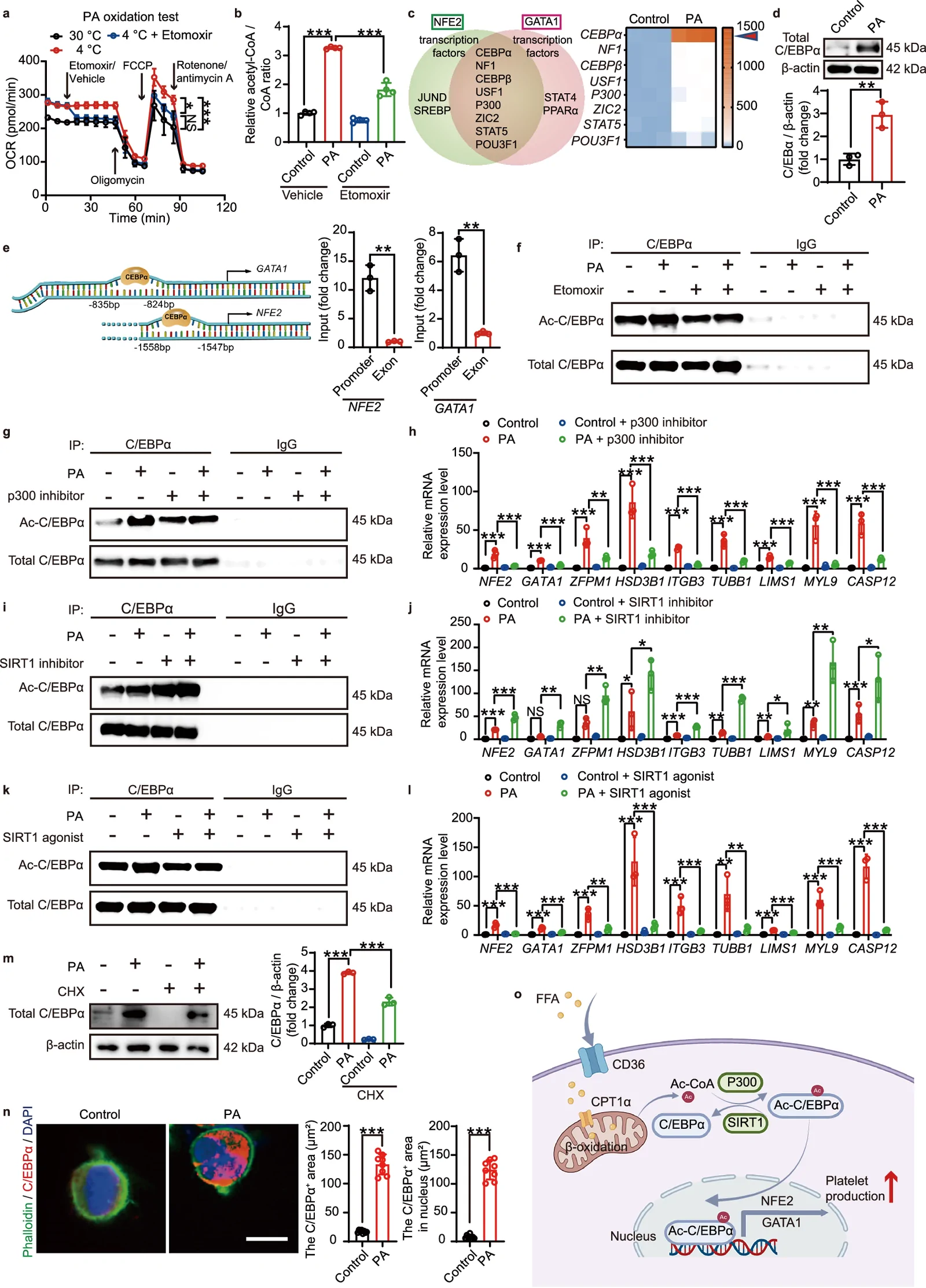
Acetyl-CoA-induced C/EBPα acetylation drives the transcription of thrombocytopoiesis-associated genes. **a** Seahorse metabolic flux assay of MKs isolated from mice under 30 °C or 4 °C and treated with PA with or without etomoxir (*n* = 6 samples per group). Oxygen consumption rate measurements were performed on cells sequentially treated with the ATP synthase inhibitor oligomycin, the uncoupler FCCP, and the RC complex I inhibitor rotenone and RC complex III inhibitor antimycin A. **b** ELISA detection of the intracellular acetyl-CoA/CoA ratio in control- or PA-treated MKs with or without etomoxir treatment (*n* = 4 samples per group). **c** Predicted TFs regulating transcription of the thrombocytopoiesis driver genes *NFE2* and *GATA1*. qPCR analysis identified *CEBPa* as the most upregulated TF gene (*n* = 3 samples per group). **d** Protein expression of C/EBPα in control- or PA-treated MKs (*n* = 3 samples per group). β-actin served as a loading control. **e** ChIP of C/EBPα binding to the *NFE2* and *GATA1* gene promoters. *NFE2* and *GATA1* exon regions served as controls (*n* = 3 samples per group). **f** Immunoprecipitation (IP) of C/EBPα in control- or PA-treated MKs with or without etomoxir administration. Acetyl-C/EBPα was detected. Non-immune IgG was used as a negative control. **g**, **i**, **k** IP of C/EBPα in control- or PA-treated MKs with or without p300 inhibitor (**g**), SIRT1 inhibitor (**i**), or SIRT1 agonist (**k**) treatment. Acetyl-C/EBPα was detected. Non-immune IgG was used as a negative control. **h**, **j**, **l** Thrombocytopoiesis-associated gene expression in control- or PA-treated MKs with or without p300 inhibitor (**h**), SIRT1 inhibitor (**j**), or SIRT1 agonist (**l**) treatment (*n* = 3 samples per group). **m** Protein expression levels of C/EBPα in DMSO control- or PA-treated MKs with or without protein synthesis inhibitor CHX (*n* = 3 samples per group). β-actin served as a loading control. **n** Control- or PA-treated MKs were stained with an anti-C/EBPα antibody (red), phalloidin (green), and DAPI (blue). Scale bar: 15 μm. Quantification of total C/EBPα^+^ signals and nuclear C/EBPα^+^ signals (*n* = 8 random fields per group). **o** Schematic model of FFA-induced platelet production. FFA β-oxidation produces acetyl-CoA for C/EBPα acetylation via the acetyltransferase p300 and deacetylase SIRT1. Acetylation enhances the stability of C/EBPα and promotes transcription of thrombocytopoiesis-associated genes (Created in BioRender. Ruibo, C. (2026) https://BioRender.com/bojdcrx). *P < 0.05; **P < 0.01; ***P < 0.001. NS not significant. Data are presented as mean ± SD.

To analyze PA metabolism in MKs, freshly isolated BM MKs from mice exposed to 30 °C or 4 °C were immediately subjected to Seahorse metabolic flux analysis. For PA metabolism analysis, a substrate-limited growth medium with reduced glucose and exogenous PA was used. As expected, MKs from cold-exposed mice had significantly higher oxygen consumption than those in the thermoneutral group (Fig. 3a). A CPT1α inhibitor completely blocked PA oxidation in freshly isolated MKs from cold-exposed mice (Fig. 3a). These findings confirm that cold exposure promotes PA oxidation in MKs. To further study the detailed signaling pathways involved in PA oxidation-induced platelet production, human MEG-01 megakaryoblastic cells, which also highly express CD36 and exhibit mature MK morphology upon PA stimulation, were used as an in vitro model (Supplementary information, Fig. S4a, b). PA-induced metabolic reprogramming in MEG-01 cells markedly increased acetyl-CoA production, and etomoxir largely reversed this effect (Fig. 3b), confirming a β-oxidation-dependent mechanism for acetyl-CoA generation.

Given that MK maturation and platelet production are regulated by a cascade of TFs, we analyzed upstream TFs that regulate *GATA1* and *NFE2* (Fig. 3c). qPCR array showed that *CEBPa* expression increased over 1000-fold in PA-stimulated MKs (Fig. 3c). Consistent with the elevated mRNA levels, PA stimulation also markedly increased C/EBPα protein levels (Fig. 3d). Chromatin immunoprecipitation (ChIP) assays demonstrated physical binding of C/EBPα to the promoter regions of *NFE2* and *GATA1* at the predicted sites (Fig. 3e).

Along with PA-induced acetyl-CoA elevation, C/EBPα acetylation increased, and this was attenuated by etomoxir (Fig. 3f). Additionally, PA stimulation upregulated p300 acetyltransferase, and treatment of MEG-01 cells with a p300 inhibitor significantly reduced acetylated C/EBPα levels (Fig. 3c, g). Consequently, the p300 inhibitor also suppressed C/EBPα-targeted *NFE2* and *GATA1* and their downstream platelet production-related genes, including *ZFPM1*, *HSD3B1*, *ITGB3*, *TUBB1*, *LIMS1*, *MYL9*, and *CASP12* (Fig. 3h). Inhibiting SIRT1 deacetylase further increased C/EBPα acetylation and the expression of platelet production-related genes (Fig. 3i, j). Conversely, activating SIRT1 with an agonist abolished PA-induced upregulation of C/EBPα and its target genes (Fig. 3k, l).

Acetylation has been reported to increase C/EBPα stability.^41^ To verify this, cycloheximide was used to inhibit protein synthesis. In the presence of cycloheximide, acetylated C/EBPα remained stable and accumulated in the nucleus (Fig. 3m, n). Notably, PA markedly upregulated NF-E2 but did not affect its acetylation, indicating that acetylation is specific to C/EBPα (Supplementary information, Fig. S4c, d). These results demonstrate that C/EBPα acetylation, regulated by p300 and SIRT1, controls the expression of platelet production-associated genes in MKs (Fig. 3o). Furthermore, the PA oxidation-induced acetyl-CoA production and C/EBPα acetylation observed in vitro were validated in isolated primary mouse MKs (Supplementary information, Fig. S4e–h). Knockdown of C/EBPα by *siCebpa* effectively abolished the PA-induced upregulation of *Nfe2* and *Gata1* (Supplementary information, Fig. S4i, j), validating the PA-acetyl-CoA-C/EBPα axis in regulating platelet production-related genes.

### Genetic deletion of adipose triglyceride lipase PNPLA2 ablates cold-induced thrombocytopoiesis

**Fig. 4:**
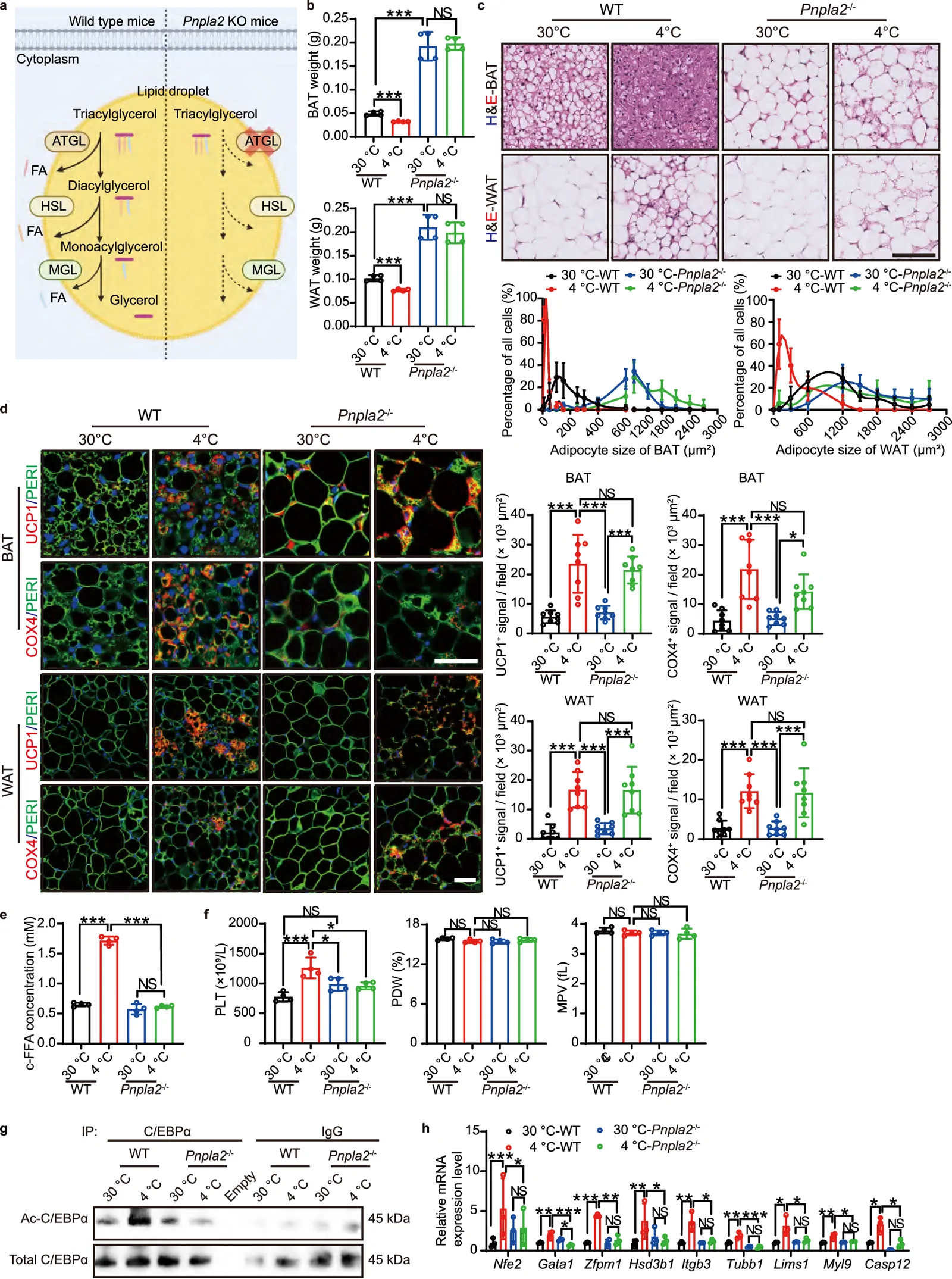
Genetic deletion of *Pnpla2* ablates cold-triggered thrombocytopoiesis. **a** Diagram of lipolysis in an adipocyte in WT mice and *Pnpla2*^*–/–*^ mice. *Pnpla2* encodes ATGL, a key intracellular lipase involved in the hydrolysis of stored fat and FFA release. *Pnpla2*^*–/–*^ mice are not able to liberate FFAs from adipose TG depots. **b** Weight quantification of BAT and WAT in WT mice and *Pnpla2*^*–/–*^ mice under 30 °C or 4 °C (*n* = 4 samples per group). **c** Histological analysis of BAT and WAT in WT mice and *Pnpla2*^*–/–*^ mice under 30 °C or 4 °C. Quantification of adipocyte size (*n* = 8 random fields per group). Scale bar: 100 μm. **d** Immunohistochemical staining of BAT and WAT with UCP1 and COX4, followed by counterstaining with DAPI (blue) in WT mice and *Pnpla2*^*–/–*^ mice under 30 °C or 4 °C. Quantification of UCP1^+^ signals and COX4^+^ signals (*n* = 8 random fields per group). Scale bar: 50 μm. **e** c-FFA levels in WT mice and *Pnpla2*^*–/–*^ mice under 30 °C or 4 °C (*n* = 4 mice per group). **f** PLT, PDW, and MPV analysis in PB from WT mice and *Pnpla2*^*–/–*^ mice under 30 °C or 4 °C (*n* = 4 mice per group). **g** IP of C/EBPα in isolated BM MKs from WT mice and *Pnpla2*^*–/–*^ mice under 30 °C or 4 °C. Acetyl-C/EBPα was detected. Non-immune IgG was used as a negative control. **h** Thrombocytopoiesis-associated gene expression in isolated MKs from WT mice and *Pnpla2*^*–/–*^ mice under 30 °C or 4 °C (*n* = 3 samples per group). *P < 0.05; **P < 0.01; ***P < 0.001. NS not significant. Data are presented as mean ± SD.

To validate the lipolysis-β-oxidation-acetylation axis in vivo, we further applied a genetic approach to delete adipose triglyceride lipase (ATGL; gene name, *Pnpla2*), a key intracellular lipase involved in the hydrolysis of stored fat, lipid partitioning, and FFA release. *Pnpla2*^*–/–*^ mice are known to have impaired ability to liberate FFAs from adipose TG depots, leading to a marked reduction in c-FFAs during fasting (Fig. 4a).^42^ Interestingly, under thermoneutrality, *Pnpla2*^*–/–*^ mice had significantly higher BAT and WAT weights than WT mice (Fig. 4b). Upon cold exposure, BAT and WAT weights remained unchanged in *Pnpla2*^*–/–*^ mice (Fig. 4b), confirming impaired lipolysis. Histological analysis further showed that adipocyte diameter did not change in cold-exposed *Pnpla2*^*–/–*^ mice (Fig. 4c). Of note, similar to WT mice, UCP1 and COX4 were upregulated in response to cold stimulation in *Pnpla2*^*–/–*^ mice (Fig. 4d), suggesting that *Pnpla2*^*–/–*^ mice retain mitochondrial function.^19^ As expected, cold-induced increases in c-FFA levels were completely abolished in *Pnpla2*^*–/–*^ mice (Fig. 4e). Correspondingly, cold-induced thrombocytopoiesis was eliminated by *Pnpla2* deletion (Fig. 4f). To validate the previously proposed molecular mechanism in vivo, we further examined C/EBPα acetylation and its downstream platelet production-related genes. Both were completely abolished by *Pnpla2* deletion (Fig. 4g, h). These results provide compelling evidence that cold-induced thrombocytopoiesis is dependent on lipolysis.

### MK-specific deletion of CPT1α ablates cold-induced thrombocytopoiesis

**Fig. 5:**
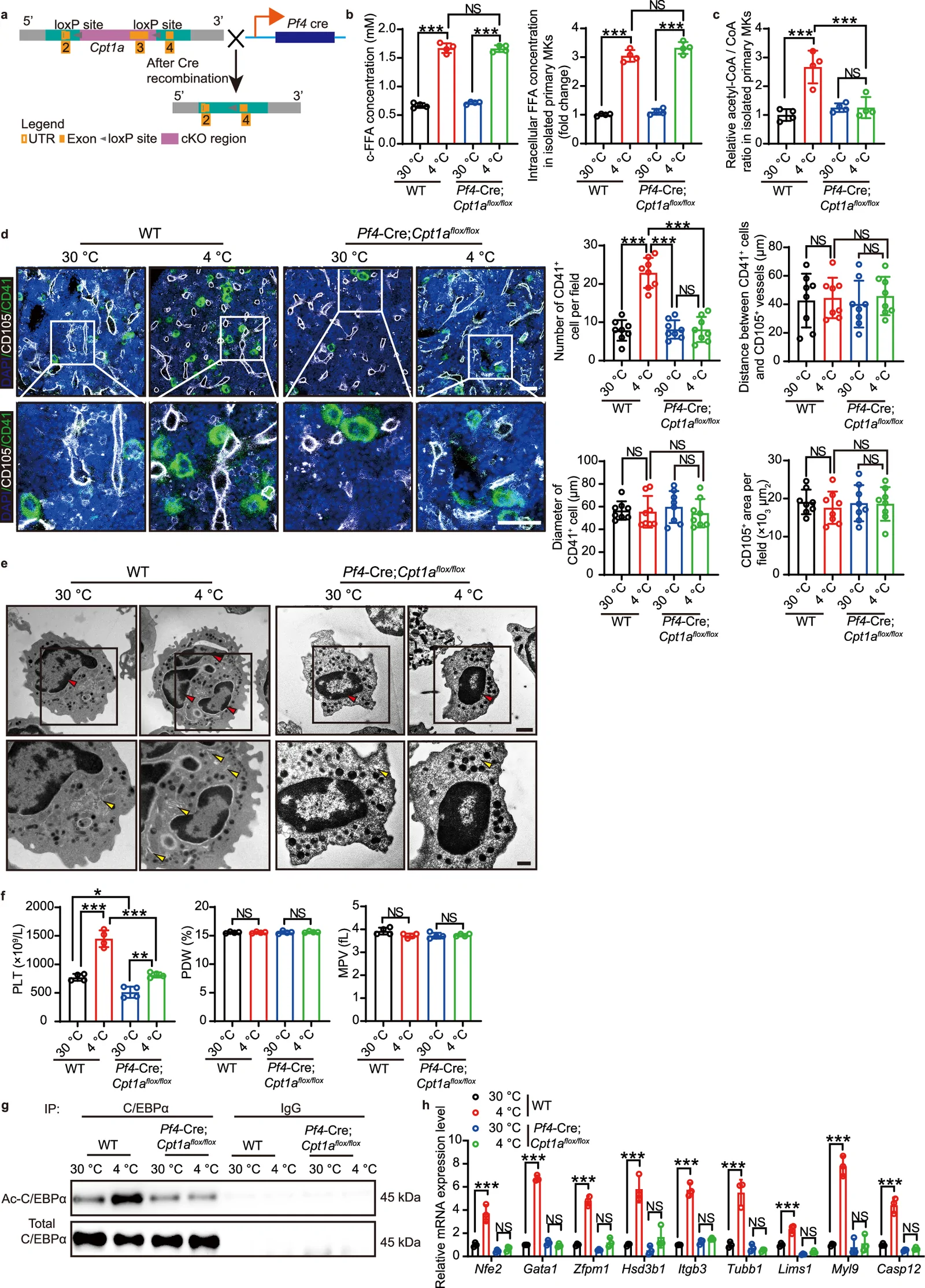
Genetic deletion of *Cpt1a* in MKs ablates cold-triggered thrombocytopoiesis. **a** Generation of *Pf4*-Cre *Cpt1a*^*flox/flox*^ mice. Mice expressing *Cre* under a cell type-specific *Pf4* promoter were crossbred with mice with loxP sites inserted into the *Cpt1a* region. MK-specific *Cpt1a* KO mice were identified by genotyping. **b** c-FFA levels in WT mice and *Pf4*-Cre *Cpt1a*^*flox/flox*^ mice under 30 °C or 4 °C (*n* = 4 mice per group). Intracellular FFA levels in BM MKs isolated from WT mice and *Pf4*-Cre *Cpt1a*^*flox/flox*^ mice under 30 °C or 4 °C (*n* = 4 mice per group). **c** ELISA detection of intracellular acetyl-CoA/CoA ratio in BM MKs isolated from WT mice and *Pf4*-Cre *Cpt1a*^*flox/flox*^ mice under 30 °C or 4 °C (*n* = 4 mice per group). **d** Representative whole-mount micrographs of CD41 (green), CD105 (white), and DAPI (blue) staining of BM from WT mice and *Pf4*-Cre *Cpt1a*^*flox/flox*^ mice under 30 °C or 4 °C. Scale bar: 100 μm. Quantification of the CD41^+^ cell number per field, the average diameter of CD41^+^ cells, the average distance between CD41^+^ cells and CD105^+^ vessels, and the CD105^+^ area per field (*n* = 8 random fields per group). **e** Representative transmission electron microscopy micrographs of MKs from WT mice and *Pf4*-Cre *Cpt1a*^*flox/flox*^ mice under 30 °C or 4 °C. Red arrowhead: nucleus. Yellow arrowheads: pre-DMS. Scale bar in upper panel: 1 μm. Scale bar in lower panel: 500 nm. **f** Analysis of PLT, PCT, PDW, and MPV in PB from WT mice and *Pf4*-Cre *Cpt1a*^*flox/flox*^ mice under 30 °C or 4 °C (*n* = 4 mice per group). **g** IP of C/EBPα in BM MKs isolated from WT mice and *Pf4*-Cre *Cpt1a*^*flox/flox*^ mice under 30 °C or 4 °C. Acetyl-C/EBPα was detected. Non-immune IgG was used as a negative control. **h** Expression of thrombocytopoiesis-associated genes in MKs isolated from WT mice and *Pf4*-Cre *Cpt1a*^*flox/flox*^ mice under 30 °C or 4 °C (*n* = 3 samples per group). *P < 0.05; **P < 0.01; ***P < 0.001. NS not significant. Data are presented as mean ± SD.

Next, we studied the role of MK CPT1α in vivo using a genetic approach to specifically delete MK CPT1α by cross-breeding MK-specific *Pf4-Cre* mice with *Cpt1a*^*flox/flox*^ mice (Fig. 5a). As expected, the c-FFA levels and intra-MK FFA levels were not affected by MK-specific *Cpt1a* deletion under cold and thermoneutral conditions (Fig. 5b). However, this approach effectively mitigated intracellular acetyl-CoA levels in MKs (Fig. 5c). Without cold stimulation, *Pf4-Cre; Cpt1a*^*flox/flox*^ mice manifested reduced platelet counts and low platelet production-associated gene expression (Supplementary information, Fig. S5a, b), with no changes in RBCs or WBCs (Supplementary information, Table S1), indicating that β-oxidation plays a homeostatic role in physiological platelet biogenesis. Cold-induced increases in CD41^+^ MKs in BM were completely abolished by MK-specific *Cpt1a* deletion (Fig. 5d). Isolation of MKs from BM validated that the cold-induced morphological changes were prevented by MK-specific *Cpt1a* deletion (Fig. 5e). Consistent with the MK findings, cold-induced thrombocytopoiesis was reduced in *Pf4-Cre; Cpt1a*^*flox/flox*^ mice (Fig. 5f). As expected, cold-induced C/EBPα acetylation and expression of platelet production-associated genes were abolished in *Cpt1a*-deficient MKs (Fig. 5g, h). These results provide compelling evidence that cold-induced thrombocytopoiesis depends on β-oxidation in MKs.

### Opposing therapeutic effects of p300 and SIRT1 in cold-induced thrombocytopoiesis

**Fig. 6:**
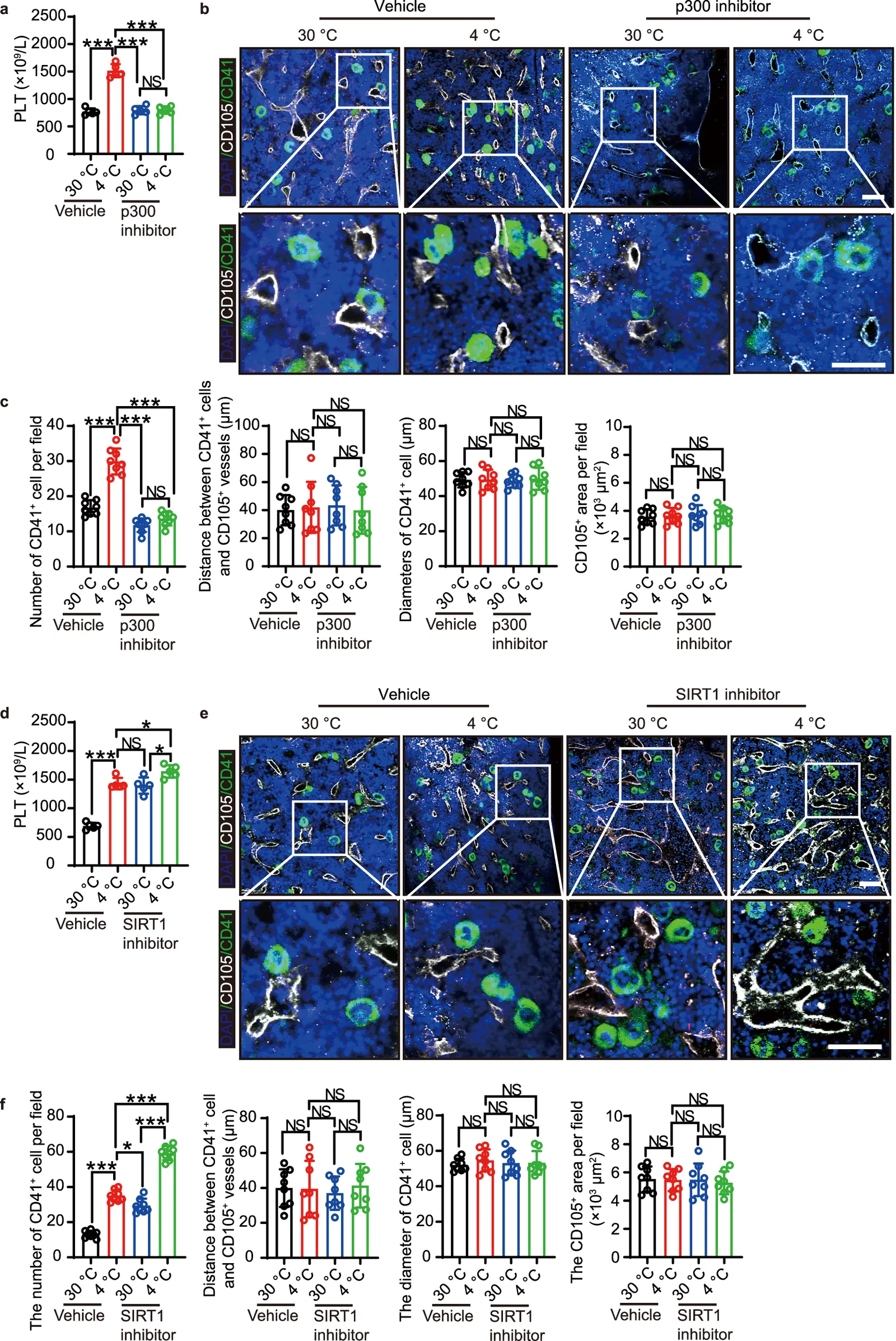
Targeting p300 or SIRT1 for modulating cold-induced thrombocytopoiesis. **a** Analysis of PLT in PB from vehicle- or p300 inhibitor-treated mice under 30 °C or 4 °C (*n* = 5 mice per group). **b** Representative whole-mount micrographs of CD41 (green), CD105 (white), and DAPI (blue) staining of BM from vehicle- or p300 inhibitor-treated mice under 30 °C or 4 °C. Scale bar: 100 μm. **c** Quantification of the CD41^+^ cell number per field, the average diameter of CD41^+^ cells, the average distance between CD41^+^ cells and CD105^+^ vessels, and the CD105^+^ area per field (*n* = 8 random fields per group). **d** Analysis of PLT in PB from vehicle- or SIRT1 inhibitor-treated mice under 30 °C or 4 °C (*n* = 5 mice per group). **e** Representative whole-mount micrographs of CD41 (green), CD105 (white), and DAPI (blue) staining of BM from vehicle- or SIRT1 inhibitor-treated mice under 30 °C or 4 °C. Scale bar: 100 μm. **f** Quantification of the CD41^+^ cell number per field, the average diameter of CD41^+^ cells, the average distance between CD41^+^ cells and CD105^+^ vessels, and the CD105^+^ area per field (*n* = 8 random fields per group). *P < 0.05; **P < 0.01; ***P < 0.001. NS not significant. Data are presented as mean ± SD.

To further investigate the roles of p300 and SIRT1 in cold-induced thrombocytopoiesis, we treated mice with specific pharmacological inhibitors under either cold exposure or thermoneutrality. Inhibiting p300 completely abolished cold-induced increases in platelet counts, with no effects on PDW or MPV (Fig. 6a; Supplementary information, Fig. S6a). Additionally, p300 inhibition reduced the expression of platelet production-related genes in MKs (Supplementary information, Fig. S6b) and decreased the number of BM MKs under cold exposure (Fig. 6b, c). These in vivo data confirm that p300 acetyltransferase is essential for cold-induced thrombocytopoiesis.

In contrast to p300, pharmacological inhibition of SIRT1 further increased platelet counts and the expression of platelet production-related genes in mice exposed to either 30 °C or 4 °C (Fig. 6d; Supplementary information, Fig. S6c, d). SIRT1 inhibition also increased the number of BM MKs (Fig. 6e, f). Notably, p300 and SIRT1 inhibitors had no effect on RBC or WBC parameters in mice under either thermoneutral or cold conditions (Supplementary information, Table S1). These findings demonstrate that acetyltransferases and deacetylases exert opposing effects on cold-induced thrombocytopoiesis, identifying them as potential therapeutic targets for regulating thrombocytopoiesis and treating thrombocytopenia.

### Cold aggravates DVT in the flow restriction mouse model

**Fig. 7:**
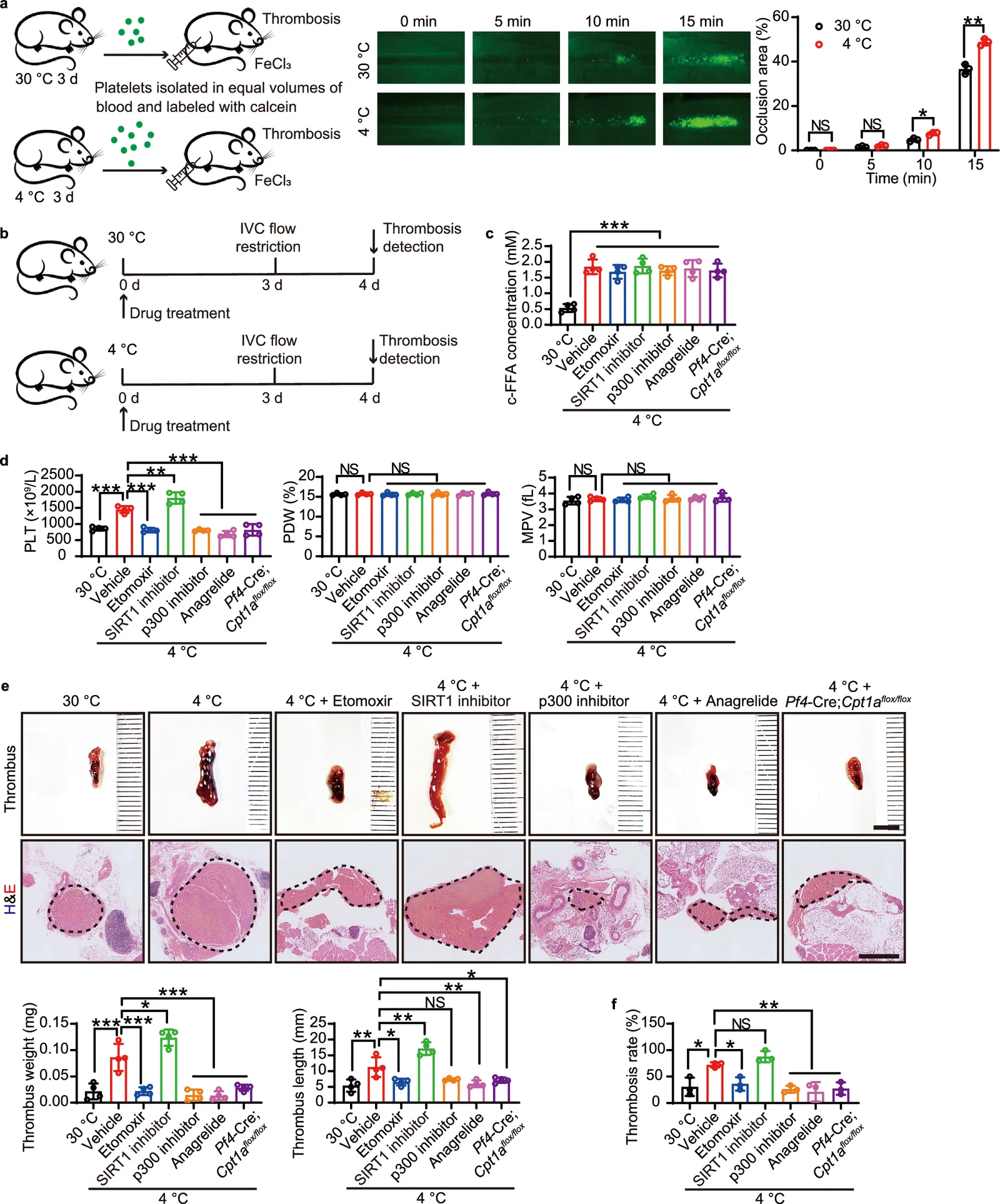
Targeting β-oxidation-acetylation for regulating cold-aggravated DVT in mice with IVC flow restriction. **a** Schematic of the thrombosis model receiving transfusion of labeled platelets in equal volumes of blood from 30 °C- and 4 °C-exposed mice. Representative thrombus micrographs upon FeCl_3_-induced endothelial injury with platelets from mice under 30 °C or 4 °C. Quantification of FeCl_3_-induced occlusion at various time points (*n* = 3 samples per group). **b** Schematic figure of the IVC flow restriction model in 30 °C- or 4 °C-exposed mice. **c** c-FFA levels in 30 °C- or 4 °C-exposed mice treated with or without the CPT1α inhibitor, the SIRT1 inhibitor, the p300 inhibitor, or platelet-lowering agents and in MK-specific *Cpt1a* KO (*n* = 4 mice per group). **d** Analysis of PLT, PDW, and MPV in PB from 30 °C- or 4 °C-exposed mice treated with or without the CPT1α inhibitor, the SIRT1 inhibitor, the p300 inhibitor, or platelet-lowering agents and in MK-specific *Cpt1a* KO (*n* = 4 mice per group). **e** Representative gross images and histological analysis of thrombi from 30 °C- or 4 °C-exposed mice treated with or without the CPT1α inhibitor, the SIRT1 inhibitor, the p300 inhibitor, or platelet-lowering agents and from MK-specific *Cpt1a* KO. Scale bar in upper panel: 5 mm. Scale bar in lower panel: 500 μm. Quantification of thrombus weight and length in various groups (*n* = 4 mice per group). **f** Quantification of thrombosis rate in various groups (*n* = 5 or 6 mice per group, statistics were performed from three independent experiments). *P < 0.05; **P < 0.01; ***P < 0.001. NS not significant. Data are presented as mean ± SD.

To investigate whether cold-induced thrombocytopoiesis contributes to thrombosis, we labeled platelets from mice exposed to either 30 °C or 4 °C with calcein and then immediately transfused them into a ferric chloride (FeCl₃)-induced vascular injury model.^43^ Thrombus formation was assessed at the injury site. When equal numbers of platelets from cold- or thermoneutral-exposed mice were transfused, thrombus formation rate and extent were indistinguishable in recipients (Supplementary information, Fig. S7a). Notably, when platelets from equal blood volumes of the two groups were transfused, cold-exposed platelets significantly increased thrombus formation rate and extent compared with thermoneutral controls (Fig. 7a), suggesting that cold-induced increases in platelet count, but not platelet activation, contribute to thrombosis in vivo.

To test whether cold-induced thrombocytopoiesis aggravates DVT, a flow restriction model of the inferior vena cava (IVC) was employed (Fig. 7b). Various pharmacological inhibitors and *Pf4-Cre; Cpt1a*^*flox/flox*^ mice were used to validate our findings in DVT. As expected, cold exposure increased c-FFA levels to about 2 mM, whereas disruption of FFA oxidation, inhibition of acetylation, or use of platelet-lowering agents did not affect c-FFA levels in this model (Fig. 7c). In this stenosis-induced DVT model, cold-induced thrombocytopoiesis was inhibited by the CPT1α inhibitor, the p300 inhibitor, platelet-lowering agents, and MK-specific *Cpt1a* deletion and was further upregulated by the SIRT1 inhibitor (Fig. 7d). None of these interventions altered PDW or MPV (Fig. 7d).

In this DVT-prone mouse model, mice commonly developed a thrombus located at the ligature (toward the foot) within 24 h after flow restriction in the IVC. Cold exposure significantly increased thrombus weight and length (Fig. 7e), and this effect was reduced by the CPT1α inhibitor, the p300 inhibitor, platelet-lowering agents, and MK-specific *Cpt1a* deletion and was exacerbated by the SIRT1 inhibitor (Fig. 7e). Following repeated experiments, the thrombosis rate was calculated. Nearly 25% of 30 °C-exposed mice and nearly 75% of cold-exposed mice developed thrombi (Fig. 7f). This rate was significantly reduced by the CPT1α inhibitor, the p300 inhibitor, platelet-lowering agents, and MK-specific *Cpt1a* deletion (Fig. 7f).

To assess the effect of cold on endothelium, we isolated IVC endothelial cells from cold- or thermoneutral-exposed mice and measured von Willebrand factor (VWF) expression, which is important for platelet adhesion and coagulation.^44^ Endothelial *Vwf* levels remained unchanged by cold exposure (Supplementary information, Fig. S7b). We also measured tissue factor levels under cold exposure and thermoneutrality in this model. Tissue factor levels showed no differences between the two groups (Supplementary information, Fig. S7c). Together, these findings demonstrate that cold aggravates DVT via the FFA-β-oxidation-C/EBPα-acetylation-thrombocytopoiesis axis in preclinical models.

### Cold aggravates RVO in mice with a laser-irradiated eye

**Fig. 8:**
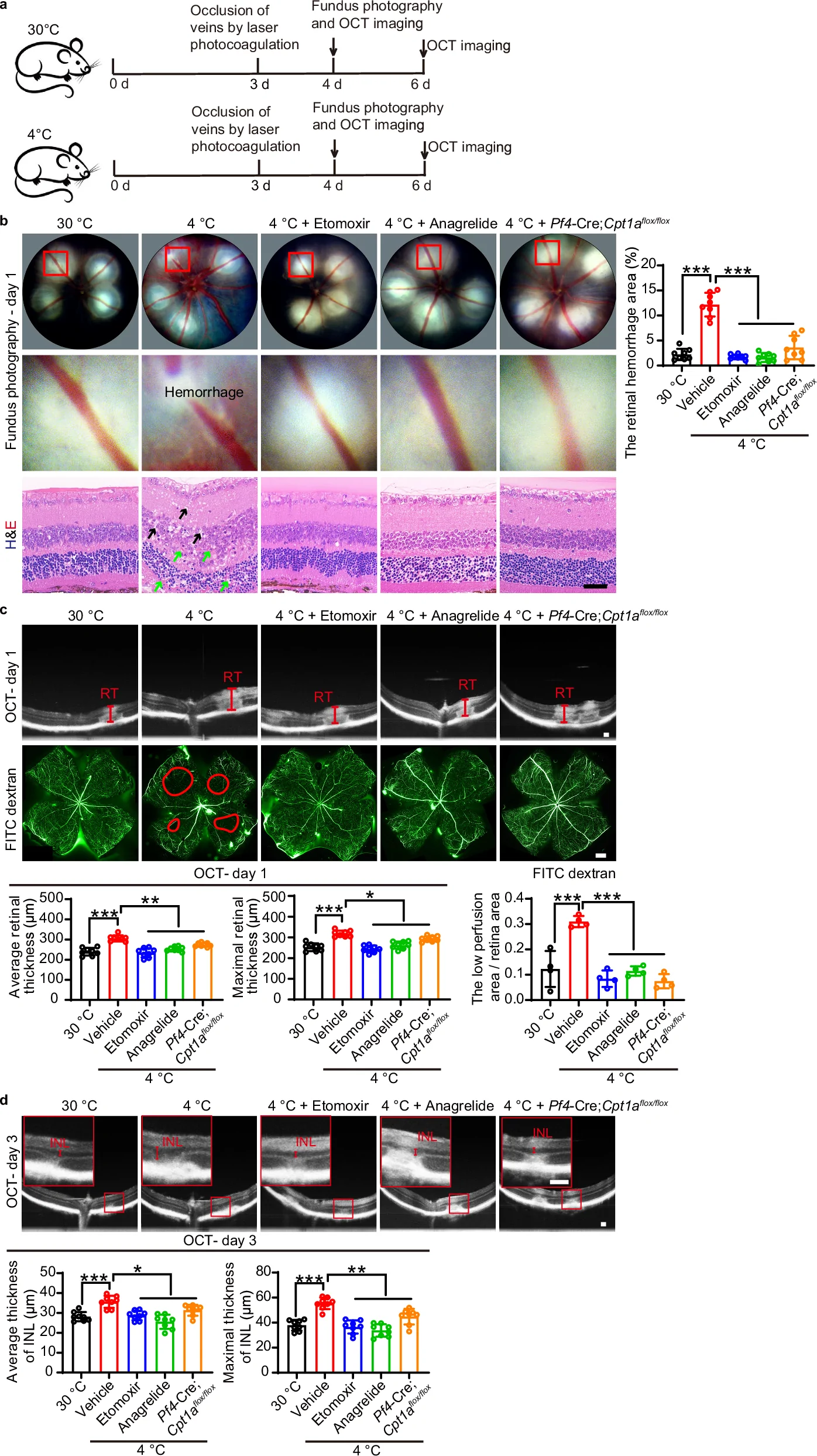
Targeting β-oxidation for treating cold-aggravated RVO. **a** Schematic of laser-induced RVO model using 30 °C- or 4 °C-exposed mice. **b** Representative fundus photography and histological analysis of 30 °C- or 4 °C-exposed mice treated with or without the CPT1α inhibitor or platelet-lowering agents and of MK-specific *Cpt1a* KO. Black arrows: cystoid edema. Green arrows: hemorrhage area. Scale bar: 50 μm. Quantification of retinal hemorrhage area in various groups (*n* = 8 eyes per group). **c** Representative OCT images of the laser-irradiated point 24 h after irradiation and retinal angiography images showing capillary perfusion in 30 °C- or 4 °C-exposed mice treated with or without the CPT1α inhibitor or platelet-lowering agents and in MK-specific *Cpt1a* KO. RT: retinal thickness. Dashed lines indicate the non-perfused capillary area. Scale bar in upper panel: 100 μm. Scale bar in lower panel: 500 μm. Quantification of retinal thickness (*n* = 8 eyes per group) and capillary perfusion in various groups (*n* = 4 eyes per group). **d** Representative OCT images of the laser-irradiated point 72 h after irradiation in 30 °C- or 4 °C-exposed mice treated with or without the CPT1α inhibitor or platelet-lowering agents and in MK-specific *Cpt1a* KO. INL: inter nuclear layer thickness. Scale bar: 100 μm. Quantification of INL thickness in various groups (*n* = 8 eyes per group). *P < 0.05; **P < 0.01; ***P < 0.001. NS not significant. Data are presented as mean ± SD.

To investigate whether cold-induced thrombocytopoiesis also contributes to small VO, we employed a mouse model of RVO induced by low-dose laser photocoagulation (Fig. 8a). Interestingly, fundus retinal imaging and histological analysis revealed significantly increased intraretinal hemorrhage and cystoid edema in cold-exposed RVO mouse models (Fig. 8b), whereas this effect was inhibited by the CPT1α inhibitor, platelet-lowering agents, and MK-specific *Cpt1a* deletion (Fig. 8b).

Optical coherence tomography (OCT), which provides real-time optical histological cross-sections of the retina, and fluorescein angiography, which visualizes non-perfusion areas of capillaries distal to the occlusion site, were used to assess the consequences of RVO. OCT and fluorescein angiography showed that 24 h after laser irradiation, cold exposure significantly increased retinal swelling and non-perfusion areas (Fig. 8c). Again, cold-induced disruption of retinal structure and the capillary network was mitigated by FFA oxidation inhibition or platelet-lowering agents (Fig. 8c). RVO-induced retinal swelling, primarily driven by edema, peaked at 24 h post-RVO and persisted for more than 3 days. At 72 h after RVO induction, the cold-exposed group still had increased thickness of the inner nuclear layer (INL), indicating that retinal edema in the INL was not alleviated (Fig. 8d).

To validate the histone acetylation mechanism in the RVO model, SIRT1 and p300 inhibitors were applied. As expected, the p300 inhibitor blocked cold-aggravated intraretinal hemorrhaging, retinal swelling, and non-perfusion areas, whereas pharmacological inhibition of SIRT1 further exacerbated these pathological effects (Supplementary information, Fig. S8a–c). Together, these results show that cold exposure aggravates small VO via the FFA-β-oxidation-C/EBPα-acetylation-thrombocytopoiesis axis in RVO models.

### Cold exposure increases c-FFA levels and platelet counts in humans

**Fig. 9:**
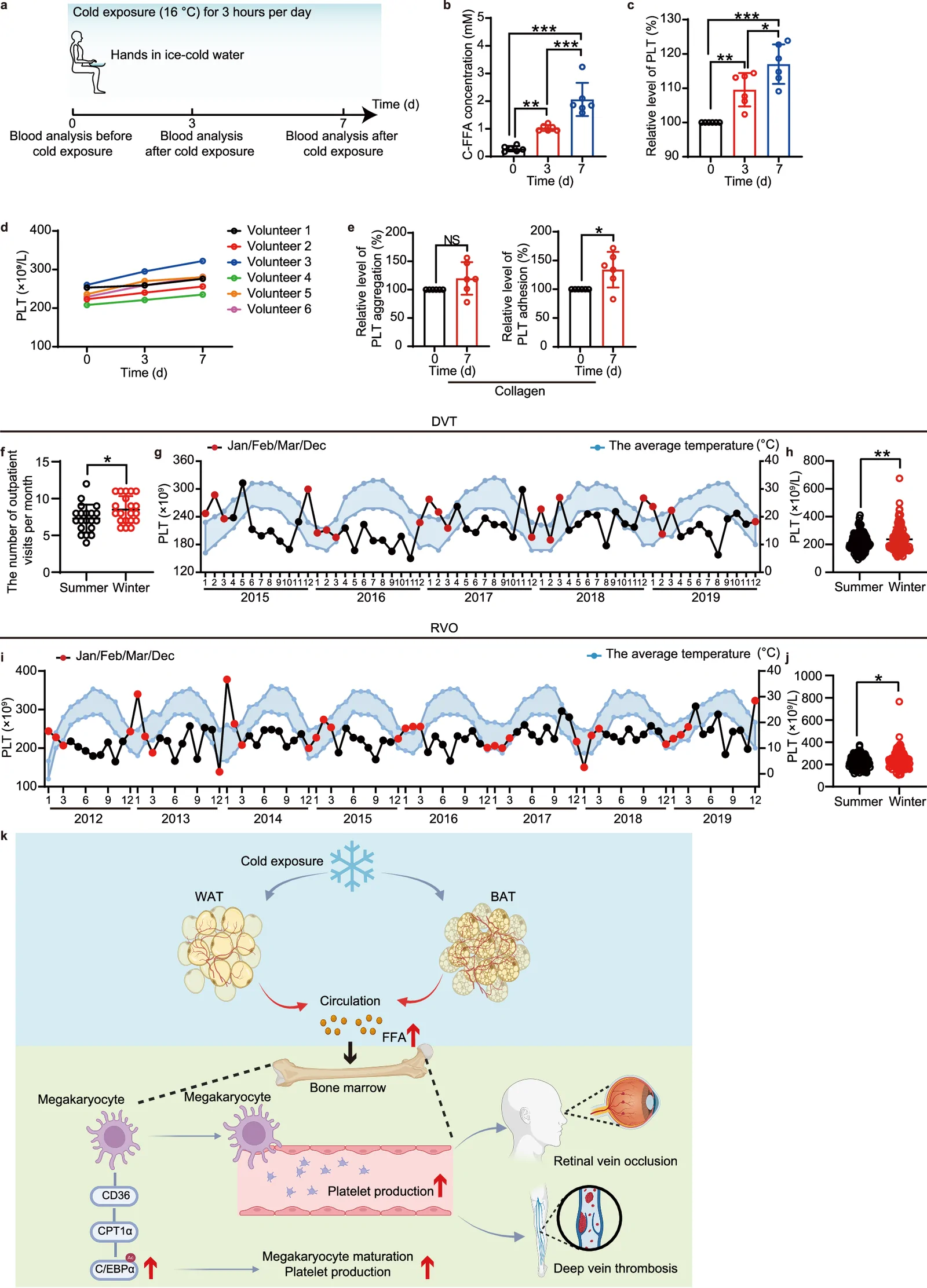
Mild cold exposure increases c-FFA levels and platelet counts in humans. **a** Schematic model of six healthy male volunteers under 16 °C cold exposure for 3 h per day. **b** c-FFA levels on days 0, 3, and 7 in volunteers under cold exposure (*n* = 6 subjects per group). **c**, **d** Fold change and individual analysis of PLT in PB on days 0, 3, and 7 from volunteers under cold exposure (*n* = 6 subjects per group). **e** Human platelet aggregometry and platelet adhesion assays in response to collagen before and after cold exposure (*n* = 6 subjects per group). **f** Treatment-naïve DVT patients (*n* = 425) from 2015–2019 were retrospectively analyzed. Number of outpatient visits per month in summer and winter seasons (20 months per group). **g** Monthly temperature fluctuations and average platelet counts of DVT patients per month during 2015–2019. Red dots: platelet counts in winter months. **h** Quantification of platelet counts of DVT patients in summer and winter seasons (*n* = 146 in summer, *n* = 164 in winter). **i** Treatment-naïve RVO patients (*n* = 448) from 2012–2019 were retrospectively analyzed. Monthly temperature fluctuations and average platelet counts of RVO patients per month during 2012–2019. Red dots: platelet counts in winter months. **j** Quantification of platelet counts of RVO patients in summer and winter seasons (*n* = 156 in summer, *n* = 127 in winter). **k** Schematic diagram showing how cold exposure increases VO risk through adipose tissue browning. The diagram shows the suggested mechanism whereby adipose tissue browning-released c-FFA stimulates BM MKs and promotes C/EBPα acetylation-triggered thrombocytopoiesis. The increased number of platelets aggravates VO, including DVT and RVO. Hence, we provide mechanistic insights into low-temperature-associated cardiovascular risks and propose a novel axis of adipose tissue NST-lipolysis-MK-platelet-VO. Targeting FFA production/transport or targeting protein acetylation in MKs are potential strategies for alleviating VO (Created in BioRender. Ruibo, C. (2026) https://BioRender.com/bojdcrx). *P < 0.05; **P < 0.01; ***P < 0.001. NS not significant. Data are presented as mean ± SD.

To study the relevance of our preclinical findings to humans, six healthy male volunteers aged 23–24 years with a BMI of 20.3–24.8 kg/m^2^ underwent tolerable cold exposure (16 °C)^45^ (Supplementary information, Table S2). These individuals were exposed to 16 °C while wearing very light clothing for 3 h per day after dinner for 7 consecutive days. Additionally, their hands were occasionally immersed in ice-cold water to further boost adipose thermogenesis.^17^ PB samples were collected on days 0, 3, and 7 (Fig. 9a) at ~22:00 after cold exposure. Our previous work reported that exposure to this cold condition enhanced BAT uptake of ^18^F-fluorodeoxyglucose (^18^F-FDG) in the supraclavicular fossa and interscapular regions, as detected by positron emission tomography-computed tomography (PET-CT).^46^ Since this cold condition activated BAT in adult humans, it was used for subsequent studies.

Exposure of humans to cold for 7 consecutive days significantly reduced BMI by approximately 2% without altering free triiodothyronine or free thyroxine hormone levels (Supplementary information, Fig. S9a, b). Cold exposure significantly increased average levels of c-FFA to approximately 2 mM on day 7 (Fig. 9b). Interestingly, average platelet counts showed an approximately 1.2-fold increase on day 7 relative to those on day 0 (Fig. 9c). Although variation existed among individuals, they all showed a trend towards increased platelet counts under this cold condition (Fig. 9d). Cold exposure had no effect on PDW, MPV, RBCs, or WBCs in humans (Supplementary information, Fig. S9c, d and Table S1). Furthermore, coagulation tests using equal numbers of platelets showed no changes in the intrinsic, extrinsic, or common coagulation pathways (Supplementary information, Fig. S9e). However, when platelets from equal blood volumes were used, 7 days of cold exposure enhanced collagen-stimulated platelet adhesion (Fig. 9e), supporting the finding that higher platelet counts aggravate clot formation. These results indicate that cold increases c-FFA levels and the number of functional circulating platelets in healthy adult humans.

### Elevated platelet counts correlate with the winter season in patients with DVT or RVO

Patients with DVT and RVO are commonly seen in cardiovascular and ophthalmology outpatient clinics, respectively. To assess the clinical relevance of our findings, we conducted a retrospective study of a cohort of 425 treatment-naïve DVT patients from 2015–2019 (Supplementary information, Table S3) at the Longyan First Hospital, located in Longyan, Fujian Province, China (25°N latitude). The median age of patients was 62 years; 154 (36.2%) were male, and 271 (63.8%) were female. The city does not provide central heating services, so residents generally do not use radiator heaters during winter. The public monthly mean high and low temperatures were obtained from the government meteorological administration website. Dec, Jan, Feb, and Mar were defined as the winter season, with average temperatures ranging from 9.3 °C to 18.6 °C. Jun, Jul, Aug, and Sep were defined as the summer season, with average temperatures ranging from 22.7 °C to 31.7 °C. The median ages of patients in winter and summer were 63 years and 61 years, respectively. Baseline characteristics were comparable in terms of age and sex (Supplementary information, Table S3).

First, we analyzed DVT patient outpatient visits and found significantly more visits in cold months than in warm months (Fig. 9f). These results are consistent with previous epidemiological reports.^4–7^ Next, we examined initial-visit platelet counts. Surprisingly, the highest monthly mean platelet counts were observed mainly in cold months (Dec 2016, Jan 2017, Mar 2018, and Jan 2019) (Fig. 9g). DVT patients in cold seasons had higher platelet counts than those in warm seasons (Fig. 9h). No seasonal differences in RBCs or WBCs were observed (Supplementary information, Table S1).

For small VO, we conducted a retrospective clinical study of 448 treatment-naïve RVO patients from 2012–2019 at the same hospital (Supplementary information, Table S4). The median age of patients was 61 years; 227 (50.7%) were male, and 221 (49.3%) were female. Again, in RVO patients, the highest monthly mean platelet counts were observed in cold months (Jan 2012, Jan 2013, Jan 2014, Feb 2015, Mar 2016, and Dec 2019) (Fig. 9i). The median ages of patients in winter and summer were 58 years and 61 years, respectively. Baseline characteristics were comparable in terms of age and sex (Supplementary information, Table S4). Similar to DVT patients, a weak but significant association was found between cold seasons and higher platelet counts in RVO patients (Fig. 9j). Our clinical data are preliminary, and the exact molecular interactions in human MKs need further verification. However, these findings are consistent with those from our preclinical models and provide evidence that cold-induced increases in platelet counts may contribute to large and small VO in humans.

## Discussion

Most geographic regions around the globe experience seasonal changes, and populations living near the northern and southern poles endure long winters every year. Numerous studies show positive correlations between cold seasons and a high prevalence of DVT and RVO in humans.^4–10^ However, the mechanisms that underlie cold-triggered high incidences of DVT and RVO remain enigmatic. Published literature reports contradictory findings regarding the effects of low environmental temperatures on platelet counts. While one study reports no alteration in platelet counts in humans exposed to low outdoor temperatures,^47^ another study with a relatively large cohort of 1095 participants demonstrates significant increases in platelet counts during exposure to low indoor temperatures.^48^ In the latter study, the authors concluded that there was a significant, independent association between lower indoor temperature and higher platelet counts in humans during winter. Although the discrepancy between these human studies remains unexplained, the fact that people wear warmer clothes outdoors and less clothing indoors may account for this observation.

In this study, we provided additional mechanistic insights at the molecular level into the increased risk of DVT and RVO during cold exposure, including increased peripheral platelet counts, a mechanistic link between NST and thrombocytopoiesis, identification of signaling pathways connecting FFA-β-oxidation to platelet production, and evidence that targeting key signaling components of the β-oxidation and acetylation pathways alleviates DVT and RVO in disease-prone models (Fig. 9k).

Since platelets play a central role in thrombosis, we focused on how cold exposure altered platelets. It is known that exposing mice to 4 °C activates BAT and induces browning in subcutaneous WAT. We demonstrated that cold-induced NST significantly contributes to thrombocytopoiesis. As major metabolites of NST-associated lipolysis, FFAs are utilized by various cell types for energy production, which is essential for cell proliferation, differentiation, and migration.^49^ We demonstrated that the FFA-β-oxidation pathway drives MK maturation and platelet production. Furthermore, our discovery may have broad implications beyond VO. For example, renal cachexia substantially induces lipolysis, and adipose atrophy has been linked to high platelet counts and high cardiovascular mortality.^50^ A similar mechanism may explain cachexia-associated cardiovascular mortality. This interesting possibility warrants further investigation.

Enhanced thrombocytopoiesis may contribute to both arterial and venous thrombosis, which are fatal processes responsible for high mortality and tissue/organ damage. It is plausible that cold exposure-triggered thrombocytopoiesis also contributes to atherosclerosis, which has a high incidence in cold seasons.^51,52^ Given that atherosclerosis involves complex etiologies, we focused on VO, which primarily involves coagulation. The adipose tissue browning-lipolysis-β-oxidation-acetylation-thrombocytopoiesis signaling axis offers opportunities for treating thrombotic diseases. Wearing warm clothing and reducing outdoor activities during cold seasons may reduce thrombotic events. Additionally, inhibition of lipolysis, β-oxidation, and acetylation by drugs offers new therapeutic opportunities for the prevention of thrombotic diseases. In our preclinical models, we provided proof-of-concept evidence of these new targets for treating thrombotic disease.

Another interesting discovery in our present study is that cold exposure increases the number of functional platelets in experimental animals and humans. Perhaps exposure to cold offers an approach for treating thrombocytopenia, a life-threatening condition associated with bleeding during surgical procedures, injury, and other medical conditions. For example, prior to surgical operations, exposure of thrombocytopenia patients to low temperatures may elevate platelet counts and prevent bleeding and hemorrhage. This therapeutic possibility warrants clinical validation.

In our pilot human study, we showed that exposure of adult humans wearing thin clothing to tolerable cold significantly increases peripheral platelet counts. Elevated platelet numbers do not directly induce VO in healthy humans or mice. In addition to elevated platelet numbers, other pathophysiological processes, such as endothelial injury, inflammation, blood velocity, blood viscosity, and coagulation factors, actively participate in venous thrombosis. Under these other pathological conditions, the increase in platelet numbers is likely to accelerate and exacerbate the formation of thrombi. Cold exposure may aggravate VO through alternative mechanisms beyond what we have proposed in the present study. Physiological cold stress is linked to glucocorticoids, catecholamines, sympathetic activation, and inflammatory cytokines, which may modulate hematopoiesis. Although we demonstrated that increased platelet numbers depend on MK FFA oxidation, we cannot exclude the potential involvement of these confounding factors. For example, it is plausible that cold exposure increases the release of catecholamines that enhance thyroid function. We showed that triiodothyronine and thyroxine remain unaltered in humans exposed to cold or thermoneutral conditions. Thus, it is unlikely that the cold exposure-catecholamine-thyroid axis significantly participates in the regulation of thrombocytopoiesis. We showed that cold exposure leads to a marked increase in maximum amplitude on thromboelastography, supporting both elevated platelet numbers and enhanced platelet activity. Although we did not observe hyperactivity of the increased platelets, subtle alterations in platelet reactivity may still contribute to thrombus formation. To generalize our findings, we reasonably speculated that other physiological and pathological conditions that increase plasma FFAs would potentially affect platelet counts. In addition to cold exposure, sympathetic activation of the β3-adrenoceptor by other mechanisms such as β3-adrenoceptor agonists would produce similar impacts on thrombocyte maturation, ultimately affecting platelet numbers. This interesting possibility warrants future investigation.

We acknowledged that our study has several limitations. (1) Human relevance. Humans are often protected from cold exposure by clothing. Although it is generally believed that the core body temperature of people living in cold geographic regions is protected by NST, these people often wear warm, thick clothing, and the average indoor temperature is relatively high. It is hard to believe that these people exhibit greater BAT activation than people living in other parts of the world. Thus, studying human relevance should consider more than the geographic factor. (2) Blood flow. In this study, we cannot exclude the potential involvement of blood velocity in thrombosis. Blood velocity is a highly dynamic factor that changes constantly over a given period of time. Thus, it is difficult to investigate its relation to thrombogenesis in our experimental settings. (3) We are uncertain about the causative links of our findings to other human diseases, including cardiovascular disease, diabetes, infectious disease, inflammatory disease, and cancer, as well as to injury. (4) In this study, we provided preliminary information relevant to drug therapies for various diseases. However, we believe that our current findings provide important clues for future drug development.

In conclusion, the data presented in our study provide mechanistic insights into cold-induced platelet production and may explain the association between cold seasons and thrombotic events. On the basis of these findings, we propose new concepts and paradigms for the possible prevention of venous thrombotic diseases to reduce mortality and improve quality of life.

## Materials and methods

### Cell isolation and cell culture

The human MEG-01 cell line was kindly provided by Dr. Xiangzhong Zhao at the central laboratory of the Affiliated Hospital of Qingdao University, Qingdao, China. Murine primary MKs were isolated from mouse BM using a mouse MK isolation kit (Cat# MAG2014M; TBD Science, Tianjin, China). Polyploidy of isolated MKs was assessed using Hoechst 33342 (1:100; Cat# H0070; Solarbio, Beijing, China) staining followed by flow cytometry (Cat# FACS Canto II; BD Biosciences, Franklin Lakes, NJ, USA). Human and mouse platelets were isolated as follows. In brief, acid-citrate-dextrose (ACD) solution was made by dissolving 85 mM sodium citrate (Cat# MB2492; Meilunbio, Dalian, China), 71.38 mM citric acid (Cat# MB8820; Meilunbio), and 27.78 mM glucose (Cat# MB2510; Meilunbio) in phosphate-buffered saline (PBS). PB samples were collected into tubes containing ACD with a volume ratio of 6:1. Platelet-rich plasma was obtained by centrifugation at 200× *g* for 10 min at 20 °C. Platelets were collected by further centrifugation at 800× *g* for 2 min at 20 °C. For primary endothelial cell isolation, a magnetic-activated cell sorting system was used (Cat# 130-042-201; Miltenyi Biotec, Bergisch Gladbach, Germany).^53^ Mouse primary MKs and mouse platelets were cultured in 10% FBS-DMEM (Cat# 40130ES76; YEASEN, Shanghai, China; Cat# MA0212-1; Meilunbio) supplemented with 100 U/mL penicillin and 100 μg/mL streptomycin (Cat# MA0110; Meilunbio). The MEG-01 cell line was cultured in 10% FBS-RPMI1640 (Cat# 40130ES76, YEASEN; Cat# MA0215-1; Meilunbio) supplemented with 100 U/mL penicillin and 100 μg/mL streptomycin. All cell lines used in our study were confirmed to be negative for mycoplasma by PCR using two primer pairs: forward: 5′-GGCGAATGGGTGAGTAACACG-3′ and reverse: 5′-CGGATAACGCTTGCGACCTATG-3′; forward: 5′-GGGAGCAAACAGGATTAGATACCCT-3′ and reverse: 5′-TGCACCATCTGTCACTCTGTTAACCTC-3′.

### Animals

All animal experiments were approved by the Animal Experimental Ethical Committee of Fudan University, Shanghai, China. Male C57BL/6 mice aged 10–13 weeks were purchased from GemPharmatech (Nanjing, China) and maintained under a 12-h light/dark cycle with food and water provided ad libitum. *Ucp1*^*–/–*^ mice on the C57BL/6 background were purchased from the Jackson Laboratory (Bar Harbor, ME, USA). *Pnpla2* Cas9-KO mice on the C57BL/6 background were purchased from GemPharmatech (Stock# T014172). To obtain MK-specific *Cpt1a* knockout mice, *Cpt1a*^*flox/flox*^ mice (Shanghai Model Organisms Center, Shanghai, China) were crossed with *Pf4-Cre* mice (Cyagen, Guangzhou, China) to obtain *Pf4-Cre Cpt1a*^*flox/flox*^ mice. Genotyping was performed using EZ DirectPCR Lysis Reagent (Cat# AN11L228; Life-iLab, Shanghai, China) followed by PCR with the following primer pairs: *Cpt1a*^*flox*^ forward: 5′-TTCTGAAGACTAGGCTAAGAGT-3′ and *Cpt1a*^*flox*^ reverse: 5′-GCCAAGGAGTAACGCACTT-3′; *Pf4-Cre* forward: 5′-CCAAGTCCTACTGTTTCTCACTC-3′ and *Pf4-Cre* reverse: 5′-TGCACAGTCAGCAGGTT-3′. All animals were randomly assigned to groups before the experiments. The experimenter was not blinded to group assignment or outcome assessment. No statistical methods were used to predetermine sample size. No samples, animals, or data were excluded.

### Human studies

All human studies were approved by the Ethical Review Committee at Longyan First Hospital Affiliated to Fujian Medical University (2021153, 2021149). Demographic information and fresh blood samples were collected from six healthy male volunteers aged 23–24 years (BMI, 20.3–24.8 kg/m^2^) with written informed consent. Prior to cold exposure, all volunteers were kept at 28 °C for 1 h. The volunteers, wearing T-shirts and shorts, were exposed to a mildly cold ambient temperature of 16 °C for 3 h per day for 7 consecutive days. During cold exposure, they occasionally placed their hands and feet in ice-cold water. Blood samples were collected for complete blood count and coagulation tests on day 0 before cold exposure and on days 3 and 7 after cold exposure. After collection, fresh platelets were immediately isolated for further investigation. On days 0 and 7 before and after cold exposure, thermal images were acquired, and BMIs were calculated (weight (kg)/(height (m))^2^). For retrospective evaluation of seasonal changes in platelet counts in patients diagnosed with DVT or RVO, the monthly mean high and low temperatures of Longyan, Fujian province, China, from 2011–2019 were downloaded from the governmental meteorological administration website. In total, 425 treatment-naïve DVT patients from 2015–2019 and 448 treatment-naïve RVO patients from 2012–2019 at the Longyan First Hospital, Longyan, Fujian, China, were included. One patient with RVO was excluded due to immune thrombocytopenia. Dec, Jan, Feb, and Mar were defined as the winter season, with average temperatures ranging from 9.3 °C to 18.6 °C. June, July, Aug, and Sep were defined as the summer season, with average temperatures ranging from 22.7 °C to 31.7 °C. Demographic information and the RBC, WBC, and platelet counts of these patients were collected for retrospective analysis.

### Cold exposure

Mice housed at room temperature were randomly divided into two groups for exposure to either 30 °C or 4 °C exposure and were housed in open cages in the climate chamber (Cat# HWS-350FT; Shanghai Binglin Electronic Technology Co., Ltd, Shanghai, China) under a 12-h light/dark cycle at the Department of Cellular and Genetic Medicine, School of Basic Medical Sciences, Fudan University. Mice were acclimated to 16 °C for one night before cold exposure at 4 °C. For the thermoneutral group, mice were maintained at 30 °C for the duration equivalent to cold exposure. For in vivo MK or platelet studies, a duration of 3 days was used for cold exposure, unless otherwise stated.

### Mouse models

For adipose tissue removal, mice were anesthetized by isoflurane (Cat# R510-22; RWD Life Science, Shenzhen, China) using a small animal anesthesia system (Cat# ALC-Ane6-V; Alcott Biotech, Shanghai, China). IBAT, iWAT, and gWAT were surgically exposed by blunt dissection and removed without compromising the blood supply to surrounding tissues. For the sham operation, adipose tissues were exposed but not excised. After wound suturing, the animals were kept at 30 °C until recovery from the operation. Mouse splenectomy was performed by creating a left subcostal surgical incision from which the spleen was removed and associated blood vessels were ligated, followed by incision closure with sterile surgical suture (Cat# CR436; Jinhuan Medical Products, Shanghai, China). For PA treatment, mice were treated with daily i.p. injections of 30 μM PA (Cat# MB7088; Meilunbio) or stearic acid (Cat# B25060; Shanghai Yuanye Biotechnology, Shanghai, China) in dimethyl sulfoxide (DMSO; Cat# PWL064; Meilunbio) for 7 days. For the mouse IVC stenosis model mimicking DVT, mice were anesthetized and placed in lateral recumbency. After gentle separation from the aorta, the IVC and side branches were ligated over a 30-gauge needle, and the needle was subsequently removed for flow restriction. After 24 h, thrombi that developed in the IVC were collected for analysis. Thrombosis rate was calculated from three independent experiments. For the FeCl_3_ injury-induced thrombosis model, platelets were isolated from 4 °C- or 30 °C-exposed mice and labeled with 1 mM calcein (Cat# 148504-34-1; Sigma-Aldrich, St. Louis, MO, USA) solution for 20 min. At the same time, male C57BL/6 mice were anesthetized, and the mesenteric vessels were exposed. FeCl_3_ injury was induced by applying filter paper saturated with 10% FeCl_3_ onto the mesenteric vessel for 2 min. After injury, calcein-labeled platelets were immediately injected intravenously. Thrombus formation started after ~5 min, and the complete thrombosis process was recorded for 15 min using fluorescence microscopy (Cat# TE2000-U; Nikon, Tokyo, Japan). For the laser-induced RVO mouse model, 5 mg/mL rose bengal (Cat# 330000; Sigma-Aldrich) was injected intravenously at a dose of 37.5 mg/kg. Mice were anesthetized by i.p. injection of 1.25% avertin (Cat# M2910; Aibei Biotechnology, Guangzhou, China), and the pupils were dilated by tropicamide (Cat# J20150137; SANTEN Pharmaceutical, Osaka, Japan). Four laser spots were created on the retinal branch veins 6 min after rose bengal injection using a 532 nm laser with a power of 80 mW, duration of 800 ms, and diameter of 50 μm (Cat# VITRA; Quantel Medical, Cournon-d’Auvergne, France). Laser spots were created at a distance from the center of the optic nerve approximately twice the diameter of the optic disc.

### Drug treatment

Isolated MKs or MEG-01 cells were cultured with 2 mM PA (Cat# MB7088; Meilunbio) in dimethyl sulfoxide (DMSO; Cat# PWL064; Meilunbio). MKs were treated with or without the CPT1α inhibitor etomoxir (50 μM; Cat# MB3404; Meilunbio), p300 inhibitor SGC-CBP30 (1 μM; Cat# MB5621; Meilunbio), SIRT1 inhibitor EX527 (5 μM; Cat# MB3761; Meilunbio), SIRT1 agonist SRT1720 (10 μM; Cat# MB3760; Meilunbio), or protein synthesis inhibitor cycloheximide (CHX; 20 μg/mL; Cat# MB2208; Meilunbio). For in vivo experiments, mice were treated with or without anagrelide hydrochloride (2 mg/kg daily, p.o.; Cat# MB1881; Meilunbio), SR59230A (20 mg/kg daily, i.p.; Cat# HY-100672; MedChemExpress, Monmouth Junction, NJ, USA), etomoxir (15 mg/kg every other day, i.p.; Cat# MB3404; Meilunbio), the SIRT1 inhibitor EX527 (50 mg/kg daily, i.p.; Cat# MB3761; Meilunbio), or the p300 inhibitor SGC-CBP30 (25 mg/kg every other day, i.p.; Cat# MB5621; Meilunbio). DMSO (Meilunbio) was used as a control.

### siRNA knockdown and overexpression

Three sets of *Cd36* siRNA (Cat#s siB13424174921, siB13424174934, and siB13424174947; RIBBIO, Guangzhou, China), *Cpt1a* siRNA (Cat#s siG171113021556, siG171113021601, and siG171113021607; RIBBIO), *Cebpa* siRNA (Cat#s siG141118162244, siG141118162315, and siG141118162406; RIBBIO), or scrambled siRNA (Cat# siN0000001-1-5; RIBBIO) were transfected into isolated MK cells using liposomal transfection reagent (Cat# 40802ES03; YEASEN). The trial that achieved the highest efficiency was selected for subsequent experiments. Knockdown efficiency was confirmed 24 h after transfection by qPCR.

### Seahorse metabolic flux assay

Fresh MKs were isolated from 30 °C- and 4 °C-exposed mice. Cellular bioenergetics were measured using a Seahorse XF Palmitate Oxidation Stress Test Kit (Cat# 103693-100; Agilent, Santa Clara, CA, USA) according to the manufacturer’s recommendations. Cells were seeded at a density of 5.0 × 10^4^ cells/well in XF DMEM supplemented with 0.5 mM glucose, 1 mM glutamine, 1% serum, and 0.5 mM carnitine. All cells were incubated overnight at 37 °C in 5% CO_2_. A Seahorse XFe96 cartridge was loaded with 150 μL XF Calibrant solution (Cat# 28318001; Agilent) per well in a separate plate and incubated overnight before measurement in a CO_2_-free environment. Cells were washed twice with substrate oxidation assay medium (XF DMEM plus 2 mM glucose and 0.5 mM carnitine) and incubated at 37 °C without CO_2_ for 60 min. Cells were treated with 0.17 mM PA in BSA solution or vehicle before the Seahorse assay. The cell plates and cartridge were then transferred to XFe 96 analyzers for assay measurement, with the sequential addition of vehicle/etomoxir (4 μM), oligomycin (1.5 μM), carbonyl cyanide 4-(trifluoromethoxy) phenylhydrazone (FCCP; 1.0 μM), and rotenone/antimycin (0.5 μM). The oxygen consumption rate (OCR) over time was recorded after sensor calibration. Metabolic parameters were calculated using XF Wave software. All experiments were performed in six replicates.

### Immunoblot, IP, and ChIP

For immunoblot analysis, equal amounts of protein samples from each group and a standard molecular weight marker (Cat# AP13L052; Life-iLab) were loaded on a 10% SDS-PAGE gel (Cat# AP15L945; Life-iLab) and transferred to a polyvinylidene difluoride (PVDF) membrane (Cat# IPVH00010; Millipore, Burlington, MA, USA), which was subsequently blocked with 5% skimmed milk for 2 h. Membranes were incubated overnight at 4 °C with primary antibodies diluted in Primary Antibody Dilution Buffer (Cat# MB9881; Meilunbio). After washing with PBS containing 0.1% Tween-20 (Cat# T8220; Solarbio), membranes were incubated at room temperature for 2 h with a goat anti-rabbit HRP-conjugated IgG antibody (1:5000; Cat# M21001L; Abmart, Shanghai, China) or a goat anti-mouse HRP-conjugated IgG antibody (1:5000; Cat# M21002L; Abmart). Target proteins were visualized via the super sensitive ECL luminescence reagent (Cat# MA0186; Meilunbio) using the Molecular Imager ChemiDoc XRS System (Bio-Rad, Hercules, CA, USA). A rabbit anti-acetyl-lysine antibody (1:1000; Cat# 502391; Zen Bio, Durham, NC, USA), a rabbit anti-C/EBPα antibody (1:1000; Cat# 18311-1-AP; Proteintech, Rosemont, IL, USA), a rabbit anti-NF-E2 antibody (1:1000; Cat# 11089-1-AP; Proteintech), and a mouse anti-β-actin antibody (1:1000; Cat# AC026; ABclonal, Wuhan, China) were used as primary antibodies. Briefly, for immunoprecipitation, ~1 × 10^5^ MKs were lysed with IP lysis buffer (50 mM Tris-HCl, pH, 7.4 (Cat# MB3739; Meilunbio; Cat# 10011018; Sinopharm Chemical Reagent Co., Ltd, Shanghai, China), 150 mM NaCl (Cat# MB2471; Meilunbio), 1 mM EDTA (Cat# MB2514; Meilunbio), 0.5% NP-40/Triton X-100 (Cat# MA0156; Meilunbio; Cat# 30188928; Sinopharm Chemical Reagent Co., Ltd), and Protease Inhibitor Cocktail (Cat# MB2678; Meilunbio)) on ice for 10 min. Lysates were cleared by centrifugation at 14,000× *g* for 10 min. Protein A/G Magnetic Beads (50 μL; Cat# HY-K0202; MedChemExpress) were conjugated to 50 μg of anti-C/EBPα antibody (Cat# 18311-1-AP; Proteintech), anti-NF-E2 antibody (Cat# 11089-1-AP; Proteintech), or IgG (Cat# AC005; ABclonal) at room temperature for 30 min. The cleared supernatants were then incubated with the antibody-bound beads and rotated at 4 °C for 2 h. Beads were collected using DynaMag-2 magnets (Cat# 12321D; Thermo Fisher Scientific, Waltham, MA, USA) and washed three times with IP wash buffer (50 mM Tris-HCl, pH, 7.4, 150 mM NaCl). Precipitated proteins were mixed with 100 μL SDS-PAGE loading buffer (Cat# MA0003-D; Meilunbio) and incubated at 95 °C for 5 min for immunoblot analysis. For the ChIP assay, a ChIP assay kit (Cat# p2078; Beyotime, Shanghai, China) was used. DNA-bound proteins were fixed using 4% paraformaldehyde (PFA; Cat# MA0192; Meilunbio). Chromatin was purified and sonicated to generate 500–1000 bp fragments. Next, 20 μL of the sonicated chromatin was collected for input, and 180 μL of the sonicated chromatin was immunoprecipitated using a rabbit anti-C/EBPα antibody (1:200; Cat# 18311-1-AP; Proteintech) or a rabbit Control IgG antibody (1:200; Cat# AC005; ABclonal). The protein–DNA complexes were mixed with 5 M NaCl and incubated at 65 °C for 6 h, and the purified DNA fraction was used for qPCR analysis. The primer pairs for ChIP are summarized in Supplementary information, Table S5. Data were normalized to non-immune rabbit IgG and are presented as mean percent input.

### RNA extraction and quantitative real-time PCR

Total RNA was extracted from adipose tissues, isolated MKs, or cultured cells using an RNAsimple Total RNA kit (Cat# DP419; TIANGEN, Beijing, China). Total RNA from each sample was reverse transcribed using Hifair II 1st Strand cDNA Synthesis SuperMix (Cat# 11123ES60; YEASEN). Reverse transcription was performed at 42 °C for 15 min, followed by enzyme inactivation at 80 °C for 5 min. The cDNA samples were subjected to qPCR using the StepOnePlus Real-Time PCR System (Applied Biosystems, Waltham, MA, USA). Each reaction was performed in triplicate in a 10 μL reaction volume containing Hieff qPCR SYBR Green Master Mix (Cat# 11203ES03; YEASEN), 50 nM forward and reverse primers, and 2 μL cDNA. The qPCR cycling conditions were 60 cycles of denaturation at 95 °C for 15 s, annealing at 60 °C for 1 min, and extension at 72 °C for 1 min. Primer pairs, including those specific for browning markers and platelet production-associated genes, are summarized in Supplementary information, Table S5.

### Histology, immunohistochemistry, immunofluorescence, and whole-mount staining

For histological analysis, adipose tissue, venous thrombi, and eyes were fixed with 4% PFA (Cat# MA0192; Meilunbio) for 24 h at room temperature. Paraffin-embedded tissues were cut into 5 µm sections, mounted onto glass slides, baked for 1 h at 60 °C, deparaffinized in xylene (Cat# 10023418; Sinopharm Chemical Reagent Co., Ltd), and sequentially rehydrated in 99%, 95%, and 70% ethanol (Cat# 10009218; Sinopharm Chemical Reagent Co., Ltd). Tissue slides were counterstained with hematoxylin (Mayer’s) (Cat# MB9897; Meilunbio) and eosin (Cat# MA0164; Meilunbio) before dehydration with 95% and 99% ethanol and were mounted with neutral balsam (Cat# 1004160; Sinopharm Chemical Reagent Co., Ltd). For immunohistochemical staining of adipose tissues, paraffin-embedded tissue sections were stained with a rabbit anti-UCP1 antibody (1:100; Cat# A5857; ABclonal) or a rabbit anti-COX4 antibody (1:100; Cat# A6564; ABclonal). After rinsing, tissue samples were incubated with a high-efficiency immunohistochemistry secondary antibody kit (Cat# abs957; Absin, Shanghai, China). For immunofluorescence staining, MKs were fixed with 4% PFA for 10 min and permeabilized with 0.1% Triton X-100 for 5 min. Cells were then blocked and stained with a primary rabbit anti-C/EBPα antibody (Cat# 18311-1-AP; Proteintech). After rinsing, samples were incubated for 45 min with a goat anti-rabbit IgG (HRP) antibody (1:4000; Cat# ab205718; Abcam, Cambridge, UK). An Alexa Fluor 555 Tyramide Super Boost Kit (Cat# B40923; Thermo Fisher Scientific) was used for antigen visualization. FITC-conjugated phalloidin (1:100; Cat# A12380; Invitrogen, Carlsbad, CA, USA) was used for co-staining. After rinsing with PBS, cells were mounted with Mounting Medium, antifading (with DAPI) (Cat# MA0236; Meilunbio). Images were captured using a HAMAMATSU microscope (Cat# C13220-0; Hamamatsu Photonics, Hamamatsu, Japan). For whole-mount staining, BM samples from femurs were decalcified for 3 days, fixed in PFA, and processed for hard tissue slicing (CryoJane; Instrumedics, Hackensack, NJ, USA). Next, 50 μm sections were blocked in PBS with 10% horse serum for 1 h, followed by staining overnight at 4 °C with a goat anti-mouse CD105 antibody (1:200; Cat# AF1320; R&D Systems, Minneapolis, MN, USA). After rinsing in PBS, blood vessels and MKs were detected using an anti-goat Alexa Fluor 647-labeled secondary antibody (1:400; Cat# A32849; Invitrogen), a rat anti-mouse CD41 antibody conjugated with FITC (1:400; Cat# 11-0411-85; Invitrogen), and DAPI (1:400; Cat# D9542; Sigma-Aldrich). After washing, samples were mounted in Vectashield mounting medium (Vector Laboratories, Newark, CA, USA) and stored at –20 °C in the dark before examination under a confocal microscope (Cat# C1; Nikon). Captured images were further analyzed using the Adobe Photoshop CS software.

### Lipidomics

Freshly obtained mouse serum samples were frozen in liquid nitrogen. Lipidomics analysis was performed with assistance from Shanghai Biotree Biotech Co., Ltd. In brief, 50 μL of mouse serum was diluted in extraction buffer (isopropanol (Cat# CAEQ-4-013493-4000; CNW Technologies GmbH, Düsseldorf, Germany) and n-hexane (Cat# CAEQ-4-011518-4000; CNW Technologies GmbH) at a 2:3 (v/v) ratio, with internal standards (Cat# CDAA252795; ANPEL, Shanghai, China)). Samples were sonicated in an ice-water bath for 5 min and then centrifuged at 4 °C for 15 min at 12,000 rpm. Next, 400 μL of supernatant was dried in a speed vac, stored under nitrogen, and reconstituted in 160 μL of n-hexane. After centrifugation at 12,000 rpm for 5 min, the supernatant was analyzed using gas chromatography-mass spectrometry (GC-MS) (Cat# 5977B; Agilent).

### Enzyme-linked immunosorbent assay

After collecting the serum and cell lysate samples, c-FFA and intracellular FFA levels were measured using an FFA ELISA kit (Cat# CK-E20817; Fanyin Biotech, Wuhan, China) according to the manufacturer’s protocol using the corresponding standard curve. Intracellular CoA and acetyl-CoA levels were measured using a CoA ELISA kit (Cat# N113721; Shanghai Jining, Shanghai, China) and an acetyl-CoA ELISA kit (Cat# N113246; Shanghai Jining). Tissue factor was measured using a tissue factor ELISA kit (Cat# E-EL-M1163; Elabscience, Wuhan, China). Absorbance values were obtained at 450 nm using a Synergy 2 Multi-Mode Microplate Reader (BioTek, Winooski, VT, USA).

### Hormone level assay

Venous blood samples were collected from patients after overnight fasting, and serum levels of free triiodothyronine and free thyroxine were measured using an electrochemiluminescence immunoassay (Cat# DXI800, Cat# A13422, and Cat# 33880; Beckman Coulter, Brea, CA, USA).

### Complete blood count and blood coagulation tests

Mouse PB samples were collected in anticoagulation tubes and analyzed using an automated hematology analyzer (Cat# BC-2600; Mindray, Shenzhen, China). Human PB samples were collected from volunteers in two separate tubes: an EDTA K2 vacuum blood collection tube (Cat# 0072497; BD Biosciences) for analysis using an automated hematology analyzer (Cat# BC-6800 PLUS; Mindray) and a sodium citrate-anticoagulation tube (Cat# 0065400; BD Biosciences) for analysis using an automated coagulation analyzer (Cat# CS-5100; Sysmex, Kobe, Japan).

### In vitro clot retraction

Clot formation was initiated by adding 300 μL platelet-rich plasma to siliconized vials containing 1 U/mL thrombin (Cat# MB1368; Meilunbio) and 20 mM CaCl_2_ (Cat# 10005861; Sinopharm, Shanghai, China). Reaction tubes were gently mixed for 5 s and then incubated at room temperature for 60 min. Clot size was measured at various time points.

### Platelet aggregometry

Turbidometric platelet aggregation was performed in platelet-rich plasma in response to 0.025 U/mL thrombin (Cat# P/N 386; Chrono-Log, Havertown, PA, USA) or 0.8 μg/mL collagen (Cat# P/N 385; Chrono-Log). Aggregation was assessed as the maximal aggregation observed 6 min after agonist challenge. Results were recorded using an aggregometer (Cat# AggRAM, Helena, Gateshead, UK). For human platelet detection, PB samples from patients were collected into sodium citrate-anticoagulation tubes (Cat# 0065400; BD Biosciences). Next, 250 μL of blood was mixed with 25 μL of platelet aggregation reagent (Collagen) (Cat# 23802100; Sinnowa, Nanjing, China). After mixing, platelet aggregation and adhesion were assessed and analyzed using an Aggrestar Platelet Function Analyzer (Cat# PL-12; Sinnowa).

### Thromboelastography

For thromboelastography, mouse PB samples were collected into sodium citrate-anticoagulation tubes (Cat# 0065400; BD Biosciences) and analyzed using a thromboelastography instrument (Cat# TEG@5000; Haemonetics Corporation, Boston, MA, USA). Reaction time (R), K time (K), α-angle (Angle), and maximum amplitude (MA) were measured.

### Flow cytometry

PB was collected and transferred to an anticoagulant tube containing ACD buffer (150 µL/mL blood). Isotonic HEPES Tyrode’s buffer was used to dilute the blood. Platelets were isolated and then incubated with a PE-conjugated anti-mouse CD62p antibody (Cat# 148305; BioLegend, San Diego, CA, USA) at room temperature for 30 min without stirring. The cell suspension was washed with ice-cold PBS and then analyzed using a FACSCanto II flow cytometer (BD Biosciences). FlowJo software (v10) was used to analyze the results. To detect immature/reticulated platelets, isolated platelets were incubated with the nucleic acid dye SYTO13 Green (Cat# S7575; Invitrogen) at room temperature for 30 min and then subjected to flow cytometry.

### Transmission electron microscopy

MKs and platelets were fixed with 2.5% glutaraldehyde in Tyrode’s buffer (Cat# DF0156; Leagene, Beijing, China) for 1.5 h at room temperature and then pelleted by centrifugation at 500× *g* for 5 min. The pellet was washed and post-fixed with 1% osmium tetroxide in Tyrode’s buffer supplemented with sucrose (25 mg/mL) for 2 h. The samples were dehydrated through a graded ethanol series, followed by treatment with acetone and propylene oxide before embedding. Polymerization was performed at 65 °C for 48 h. Ultrathin sections were prepared using an Ultracut E system (Leica, Wetzlar, Germany) and stained with uranyl acetate and lead citrate. The specimens were examined using a Tecnai G2 Spirit electron microscope (FEI, Eindhoven, Netherlands).

### Database analysis

*Cd36* expression and *Cpt1a* mRNA expression levels in various blood cells were collected from the single-cell RNA-seq datasets in Atlas of Mouse Blood Cells.^54^ For TF prediction, the 2000 bp promoters of *NFE2* and *GATA1* were analyzed using the online tool PROMO.^55^

### Fundus photography and OCT

After laser irradiation for 24 h or 72 h, mice were anesthetized, and the pupils were dilated. Fundus photography was performed using a fundus microscopic imaging system (Cat# OPTO-RIS; Optoprobe, Burnaby, Canada). OCT images were captured by an ultramicro ophthalmic imaging system (Cat# ISOCT; Optoprobe). Vertical OCT scans were performed at the RVO occlusion point. OCT images were analyzed, and the thicknesses of the retina and INL at the occlusion point were measured.

### Retinal capillary perfusion analysis

To measure the perfusion of retinal vasculature, the mice were injected intravenously with 250 μL of 40 mg/mL fluorescein-conjugated dextran with a molecular weight of 2000 kDa (Cat# FD2000S; Sigma-Aldrich). After 15 min, the mice were sacrificed, and the eyes were enucleated and fixed in 4% PFA for 1 h. The retina was then isolated from the eyecup, flat-mounted on a glass slide, and imaged using a fluorescence microscope (Cat# DMI8; Leica).

### Statistical analysis

Statistical analysis was performed using GraphPad Prism (GraphPad, USA). The normality of data was tested by the D’Agostino-Pearson normality test. Statistical differences between two groups were determined by a two-tailed Student’s *t-*test. *P* < 0.05 was considered statistically significant, *P* < 0.01 was very significant, and *P* < 0.001 was extremely significant. Differences among multiple groups were evaluated using a one-way ANOVA test. For evaluating the impact of two independent factors on a dependent variable, two-way ANOVA test was used. The data is presented as mean ± SD.

## Supplementary information

Supplementary Fig. S1

Supplementary Fig. S2

Supplementary Fig. S3

Supplementary Fig. S4

Supplementary Fig. S5

Supplementary Fig. S6

Supplementary Fig. S7

Supplementary Fig. S8

Supplementary Fig. S9

Supplementary Table S1

Supplementary Table S2

Supplementary Table S3

Supplementary Table S4

Supplementary Table S5

## Data Availability

All data of lipidomics have been deposited in EMBL-EBI’s MetaboLights (MTBLS14667).
