# Supplementary material for: Cold exposure aggravates vein occlusion through non-shivering thermogenesis-induced thrombocytopoiesis
Source: Cell Res. 2026 Jul 30;36(9):698–717. doi: 10.1038/s41422-026-01280-2 (PMC13503729; doi:10.1038/s41422-026-01280-2)
Supplement: Supplementary file 1 [file 41422_2026_1280_MOESM10_ESM.pdf]

Table S1. Complete blood test results of various groups

			RBC ($\times 10^{12}$/L)	HGB (g/L)	HCT (%)	RDW (%)	WBC ($\times 10^{9}$/L)	LYM ($\times 10^{9}$/L)	MON ($\times 10^{9}$/L)	NEU ($\times 10^{9}$/L)
**Cold exposure in wt mice for 1/12 d**	Mean ± SD	30°C (A)	9.08±0.64	128.83±7.36	41.68±2.87	14.75±0.59	3.40±1.37	2.32±0.91	0.12±0.08	0.97±0.67
		4°C (B)	9.19±0.46	131.33±3.61	41.88±0.71	14.65±0.62	4.50±1.73	3.33±1.30	0.12±0.08	1.05±0.39
	p value	A-B	0.7407	0.4724	0.8719	0.7802	0.2489	0.1472	>0.9999	0.7971
	n	/	6	6	6	6	6	6	6	6
**Cold exposure in wt mice for 1 d**	Mean ± SD	30°C (C)	8.89±0.21	122.33±9.35	40.48±2.24	14.77±1.35	3.42±1.08	2.37±0.69	0.08±0.04	0.97±0.39
		4°C (D)	8.96±0.46	130.00±4.73	41.27±2.36	14.37±0.79	2.70±1.61	1.85±1.21	0.08±0.08	0.77±0.40
	p value	C-D	0.7184	0.1035	0.5689	0.5463	0.3857	0.3856	>0.9999	0.3991
	n	/	6	6	6	6	6	6	6	6
**Cold exposure in wt mice for 2 d**	Mean ± SD	30°C (E)	9.50±0.37	133.83±6.43	41.97±4.57	14.70±0.47	4.70±1.75	2.97±0.88	0.17±0.08	1.57±1.10
		4°C (F)	9.57±0.67	135.67±10.46	42.02±5.10	14.93±0.62	3.52±1.61	2.48±1.14	0.20±0.14	0.83±0.42
	p value	E-F	0.8191	0.7222	0.9861	0.4813	0.2505	0.4304	0.6279	0.1580
	n	/	6	6	6	6	6	6	6	6
**Cold exposure in wt mice for 3 d**	Mean ± SD	30°C (G)	9.23±0.45	129.67±6.53	41.42±3.80	15.00±0.68	4.48±1.14	3.38±1.01	0.12±0.04	0.98±0.24
		4°C (H)	8.59±0.83	123.17±5.12	39.98±2.29	15.77±1.46	3.88±1.59	2.28±0.90	0.32±0.24	1.28±0.60
	p value	G-H	0.1268	0.0839	0.4468	0.2697	0.4712	0.0748	0.0720	0.2851
	n	/	6	6	6	6	6	6	6	6
**Cold exposure in wt mice for 7 d**	Mean ± SD	30°C (I)	9.04±0.29	121.17±6.40	40.00±3.25	14.98±1.03	5.42±2.93	3.92±2.27	0.18±0.10	1.32±0.63
		4°C (J)	9.50±0.74	131.17±11.18	42.48±3.82	14.92±1.27	4.33±1.93	3.17±1.41	0.17±0.18	1.00±0.43
	p value	I-J	0.1889	0.0864	0.2525	0.9223	0.4677	0.5081	0.8430	0.3331
	n	/	6	6	6	6	6	6	6	6
**Cold exposure in wt mice for 14 d**	Mean ± SD	30°C (K)	9.04±0.68	129.67±9.75	41.43±3.72	14.63±1.14	4.53±2.07	3.23±1.88	0.13±0.05	1.15±0.36
		4°C (L)	9.37±1.21	130.17±16.47	41.57±6.08	14.95±1.49	4.17±2.16	2.88±1.54	0.13±0.08	1.15±0.59
	p value	K-L	0.5784	0.9502	0.9643	0.6881	0.7701	0.7316	>0.9999	>0.9999
	n	/	6	6	6	6	6	6	6	6
**PA-treated wt mice**	Mean ± SD	Vehicle (M)	8.37±0.39	119.20±4.92	35.06±2.70	14.66±0.38	3.52±0.78	2.00±0.41	0.14±0.05	1.38±0.33
		PA (N)	8.33±0.43	118.80±5.93	34.14±4.91	14.70±0.34	3.80±0.76	2.36±0.63	0.12±0.04	1.32±0.26
	p value	M-N	0.8755	0.9105	0.7230	0.8646	0.5805	0.3126	0.5447	0.7593
	n	/	5	5	5	5	5	5	5	5
***Pf4*-Cre; *Cpt1*$^{flox/flox}$ mice vs wt mice**	Mean ± SD	wt (O)	7.82±0.60	120.67±6.35	37.00±2.34	15.60±0.72	4.43±1.56	3.07±1.22	0.13±0.06	1.23±0.50
		Pf4-Cre; *Cpt1*$^{flox/flox}$ (P)	7.95±0.69	122.67±4.04	37.57±2.37	15.17±1.18	4.53±0.67	3.47±0.61	0.1±0.00	0.97±0.06
	p value	O-P	0.8277	0.6693	0.7829	0.6171	0.9234	0.6388	0.3739	0.4135
	n	/	3	3	3	3	3	3	3	3
**Cold exposure in *Pf4*-Cre; *Cpt1*$^{flox/flox}$ mice and wt mice**	Mean ± SD	30°C wt (Q)	8.56±0.20	130.25±5.91	38.35±0.58	14.48±0.93	4.10±0.70	2.80±0.74	0.10±0.00	1.20±0.32
		4°C wt (R)	8.72±0.32	128.75±6.75	38.05±0.89	14.53±1.02	4.30±1.51	2.90±0.84	0.13±0.05	1.18±0.84
		30°C *Pf4*-Cre; *Cpt1*$^{flox/flox}$ (S)	8.52±0.43	129.25±4.35	38.18±0.39	14.35±0.68	3.58±1.91	2.48±1.26	0.15±0.06	0.95±0.61
		4°C *Pf4*-Cre; *Cpt1*$^{flox/flox}$ (T)	8.55±0.39	128.75±6.45	38.38±0.46	14.18±0.76	4.23±1.38	3.03±0.98	0.13±0.05	1.08±0.36

	p value	Q-R	0.4046	0.7495	0.5924	0.9447	0.8180	0.8641	0.3559	0.9575
		S-T	0.9208	0.9019	0.5325	0.7423	0.6019	0.5163	0.5370	0.7372
	n	/	4	4	4	4	4	4	4	4
**SIRT1 inhibitor-treated wt mice (30°C/4°C)**	Mean ± SD	30°C + vehicle (U)	8.61±0.34	130.80±4.60	39.04±0.94	14.20±1.27	3.90±1.62	2.94±1.16	0.12±0.04	0.84±0.44
		4°C + vehicle (V)	8.86±0.28	133.40±7.06	39.22±3.16	14.60±1.02	4.22±1.61	3.12±1.72	0.12±0.04	0.98±0.31
		30 °C + SIRT1 inhibitor (W)	8.72±0.26	131.20±6.10	39.23±3.36	14.54±1.27	4.72±1.37	3.76±1.02	0.12±0.04	0.84±0.30
		4 °C + SIRT1 inhibitor (X)	8.70±0.21	133.40±6.69	38.40±2.62	14.44±1.49	3.66±1.67	2.48±1.40	0.12±0.04	1.06±0.29
	p value	U-V	0.2285	0.5097	0.9058	0.5987	0.7625	0.8509	>0.9999	0.5802
		W-X	0.9079	0.6018	0.6746	0.9120	0.3042	0.1370	>0.9999	0.2747
	n	/	5	5	5	5	5	5	5	5
**p300 inhibitor-treated wt mice (30°C/4°C)**	Mean ± SD	30°C + vehicle (Y)	8.70±0.29	130.80±2.39	39.54±1.53	14.28±1.19	4.22±1.65	2.92±1.06	0.18±0.13	1.12±0.49
		4°C + vehicle (Z)	8.69±0.49	130.80±7.98	37.58±2.93	14.46±0.99	3.80±1.55	2.24±1.00	0.16±0.09	1.40±0.52
		30 °C + p300 inhibitor (AA)	8.66±0.32	129.00±7.52	39.34±2.24	14.32±1.32	4.52±1.29	3.24±1.07	0.14±0.05	1.14±0.22
		4 °C + p300 inhibitor (AB)	8.60±0.42	127.40±7.23	39.88±1.88	14.58±1.14	4.22±1.12	2.96±0.75	0.12±0.04	1.14±0.34
	p value	Y-Z	0.9696	>0.9999	0.2214	0.8014	0.6895	0.3263	0.7845	0.4071
		AA-AB	0.8135	0.7404	0.6902	0.7469	0.7046	0.6455	0.5447	>0.9999
	n	/	5	5	5	5	5	5	5	5
**Volunteers exposed to tolerable cold (0/3/7 d)**	Mean ± SD	0 (AC)	5.42±0.75	154.50±9.40	51.80±6.43	14.40±2.20	6.98±1.30	2.98±0.24	0.43±0.11	3.66±1.06
		3 (AD)	5.31±0.85	150.50±9.75	44.83±2.58	12.73±1.20	7.63±1.77	2.87±0.56	0.52±0.13	4.12±1.40
		7 (AE)	5.44±0.79	153.83±9.35	45.75±2.34	12.65±1.16	7.85±1.73	2.72±0.41	0.51±0.11	4.61±1.80
	p value	AC-AD	0.8120	0.4860	0.0335	0.1342	0.4797	0.6700	0.2205	0.5362
		AC-AE	0.9621	0.9044	0.0555	0.1157	0.3469	0.2138	0.2465	0.2913
		AD-AE	0.7811	0.5590	0.5334	0.9049	0.8381	0.6111	0.8680	0.6095
	n	/	6	6	6	6	6	6	6	6
**Patients with DVT (Summer/ Winter)**	Mean ± SD	Summer (AF)	4.20±0.72	/	/	/	7.75±3.44	/	/	/
		Winter (AG)	4.15±0.72	/	/	/	7.92±3.08	/	/	/
	p value	AF-AG	0.5299	/	/	/	0.6399	/	/	/
	n (AF)	/	146	/	/	/	146	/	/	/
	n (AG)	/	170	/	/	/	169[a]	/	/	/

[a] One data was missing.
