# Supplementary material for: Cold exposure aggravates vein occlusion through non-shivering thermogenesis-induced thrombocytopoiesis
Source: Cell Res. 2026 Jul 30;36(9):698–717. doi: 10.1038/s41422-026-01280-2 (PMC13503729; doi:10.1038/s41422-026-01280-2)
Supplement: Supplementary file 2 [file 41422_2026_1280_MOESM11_ESM.pdf]

Table S2. **Baseline information of the volunteers**

Number	Age	Gender	Height (cm)	Weight (Kg)	BMI (kg/m^2)
1	23	Male	173	70	23.4
2	24	Male	159	58	22.9
3	23	Male	172	60	20.3
4	24	Male	170	60	20.8
5	23	Male	170	63	21.8
6	24	Male	183	83	24.8
