# Supplementary material for: Cold exposure aggravates vein occlusion through non-shivering thermogenesis-induced thrombocytopoiesis
Source: Cell Res. 2026 Jul 30;36(9):698–717. doi: 10.1038/s41422-026-01280-2 (PMC13503729; doi:10.1038/s41422-026-01280-2)
Supplement: Supplementary file 3 [file 41422_2026_1280_MOESM12_ESM.pdf]

Table S3. **Baseline characteristics of the patients with DVT**

Diagnosis season	Winter (W)	Summer (S)	All seasons	*p*-value
**Gender**	*n* = 170	*n* = 146	*n* = 425	
**Male, no. (%)**	57 (33.5)	58 (39.8)	154 (36.2)	
**Female, no. (%)**	113 (66.5)	88 (60.2)	271 (63.8)	
**Age**				
**Median, no. (range)**	63 (17-100)	61 (25-91)	62 (17-100)	0.2019 (W vs S)
