# Supplementary material for: Cold exposure aggravates vein occlusion through non-shivering thermogenesis-induced thrombocytopoiesis
Source: Cell Res. 2026 Jul 30;36(9):698–717. doi: 10.1038/s41422-026-01280-2 (PMC13503729; doi:10.1038/s41422-026-01280-2)
Supplement: Supplementary file 4 [file 41422_2026_1280_MOESM13_ESM.pdf]

Table S4. **Baseline characteristics of the patients with RVO**

Diagnosis season	Winter (W)	Summer (S)	All seasons	*p*-value
**Gender**	*n* = 134	*n* = 159	*n* = 448	
**Male, no. (%)**	63 (47.1)	81 (52.7)	227 (50.7)	
**Female, no. (%)**	71 (52.9)	78 (47.3)	221 (49.3)	
**Age**				
**Median, no. (range)**	58 (23-85)	61 (13-101)	61 (13-101)	0.1139 (W vs S)
