# Supplementary material for: Cold exposure aggravates vein occlusion through non-shivering thermogenesis-induced thrombocytopoiesis
Source: Cell Res. 2026 Jul 30;36(9):698–717. doi: 10.1038/s41422-026-01280-2 (PMC13503729; doi:10.1038/s41422-026-01280-2)
Supplement: Supplementary file 5 [file 41422_2026_1280_MOESM14_ESM.pdf]

Table S5. Primer pairs used in qPCR and ChIP

Gene	Forward primer (5'- 3')	Reverse primer (5'- 3')
mouse *Nfe2*	TCCTCAGCAGAACAGGAACAG	GGCTCAAAAGATGTCTCACTTGG
human *NFE2*	GCAGGAACAGGGTGATACAGC	GCAGCTCGGTGATGGACAT
mouse *Gata1*	TGGGGACCTCAGAACCCTTG	GGCTGCATTTGGGGAAGTG
human *GATA1*	CTGTCCCCAATAGTGCTTATGG	GAATAGGCTGCTGAATTGAGGG
mouse *Zfpm1*	AGGAAACAGAGCAATCCCCG	CAGGTGGGCTCACATCTTCT
human *ZFPM1*	CGTGCTTCGAGTGCGAGAT	GGCCTGAACAGTAGAGGCG
mouse *Hsd3b1*	TGGACAAAGTATTCCGACCAGA	GGCACACTTGCTTGAACACAG
human *HSD3B1*	CACATGGCCCGCTCCATAC	GTGCCGCCGTTTTTCAGATTC
mouse *Itgb3*	CCACACGAGGCGTGAACTC	CTTCAGGTTACATCGGGGTGA
human *ITGB3*	GTGACCTGAAGGAGAATCTGC	CCGGAGTGCAATCCTCTGG
mouse *Tubb1*	CTGGGAGGTGATCGGGGAA	GCACATACTTCTTACCGTAGGCT
human *TUBB1*	AACACGGGATCGACTTGGC	CTCGGGGCACATATTTCCTAC
mouse *Lims1*	CAGCGTCCTTGAAGCTAATGG	TTACTGTTCACGATCTTCTCAGC
human *LIMS1*	TGGCCGAGTTATCAAAGCCAT	CTTTCTCACGATTATGACAGGGG
mouse *Myl9*	ACAGCGCCGAGGACTTTTC	AGACATTGGACGTAGCCCTCT
human *MYL9*	TCTTCGCAATGTTTGACCAGT	GTTGAAAGCCTCCTTAAACTCCT
mouse *Casp12*	AGACAGAGTTAATGCAGTTTGCT	TTCACCCCACAGATTCCTTCC
human *CASP12*	AACAACCGTAACTGCCAGAGT	CTGCACCGGCTTTTCCACT
mouse *Actb*	GGCTGTATTCCCCTCCATCG	CCAGTTGGTAACAATGCCATGT
human *ACTB*	CATGTACGTTGCTATCCAGGC	CTCCTTAATGTCACGCACGAT
mouse *Prdm16*	CCAAGGCAAGGGCGAAGAA	AGTCTGGTGGGATTGGAATGT
mouse *Ucp1*	ACTGCCACACCTCCAGTCATT	CTTTGCCTCACTCAGGATTGG
mouse *Pparg*	TCGCTGATGCACTGCCTATG	GAGAGGTCCACAGAGCTGATT
mouse *Cidea*	TGACATTCATGGGATTGCAGAC	GGCCAGTTGTGATGACTAAGAC
mouse *Cox4*	ATTGGCAAGAGAGCCATTTCTAC	CACGCCGATCAGCGTAAGT
mouse *Cpt1α*	CTCCGCCTGAGCCATGAAG	CACCAGTGATGATGCCATTCT
mouse *Cd36*	ATGGGCTGTGATCGGAACTG	GTCTTCCCAATAAGCATGTCTCC

human ***CD36***	AAGCCAGGTATTGCAGTTCTTT	GCATTTGCTGATGTCTAGCACA
mouse ***Cebpa***	CAAGAACAGCAACGAGTACCG	GTCACTGGTCAACTCCAGCAC
human ***CEBPα***	AGGAGGATGAAGCCAAGCAGCT	AGTGCGCGATCTGGAACTGCAG
human ***CEBPβ***	CTTCAGCCCGTACCTGGAG	GGAGAGGAAGTCGTGGTGC
human ***USF1***	TCCCAGACTGCTCTATGGAGA	CGGTGGTTACTCTGCCGAAG
human ***NF1***	GCTTTCGTATAAGCCCTCACAA	GCGGAATTGGTGATGATTCGATG
human ***STAT5***	CAGTGGTTTGACGGGGTGAT	GTCGTGGGCCTGTTGCTTAT
human ***P300***	TTCCCCTAACCTCAATATGGGAG	GCCTGTGTCATTGGGCTTTTG
human ***ZIC2***	GCGCAACTCCACAACCAGTA	TGCCGCATATAGCGGAAAAAG
human ***POU3F1***	CGCTCTACGGTAACGTGTTCT	CCAAGCCGGTGATCTCGTG
mouse ***Runx1***	ACGATGAAAACTACTCGGCAG	CTGAGGTCGTTGAATCTCGCT
mouse ***Fli1***	GCAGTGTCAGCAAATGTAACAAG	GGGCCGTTCTTCTCATCCAT
mouse ***Gfi1b***	CTTCCACCAGAAGTCGGACAT	CTTCCACCAGAAGTCGGACAT
mouse ***Mecom***	AAGTAATGAGTGTGCCTATGGC	AGTTGACTCTCGAAGCTCAAAC
mouse ***Etv6***	AGCAGGAACGAATTTCATACACG	GGCAGGTGGATCGAGTCTTC
mouse ***vWF***	CTCTTTGGGGACGACTTCATC	TCCCGAGAATGGAGAAGGAAC
human ***NFE2*** promoter	TGTTTGACCTGGGGCTTTGGT	GTGTCCTTTCACCTGTAGCCTTC
human ***NFE2*** exon	TAGAGCCATCTGGGCTTTCCG	TGCCAAGTCAGTTCCATCTCT
human ***GATA1*** promoter	AGGGCTATGTACTCTCCCTTGTAT	AAGACACCTCTGACTTAAGTGGC
human ***GATA1*** exon	GTTAATCCCCAGAGGCTCCA	CGTCCCTGTAGTAGGCCAGT
