# Supplementary material for: Cold exposure aggravates vein occlusion through non-shivering thermogenesis-induced thrombocytopoiesis
Source: Cell Res. 2026 Jul 30;36(9):698–717. doi: 10.1038/s41422-026-01280-2 (PMC13503729; doi:10.1038/s41422-026-01280-2)
Supplement: Supplementary file 6 [file 41422_2026_1280_MOESM1_ESM.pdf]

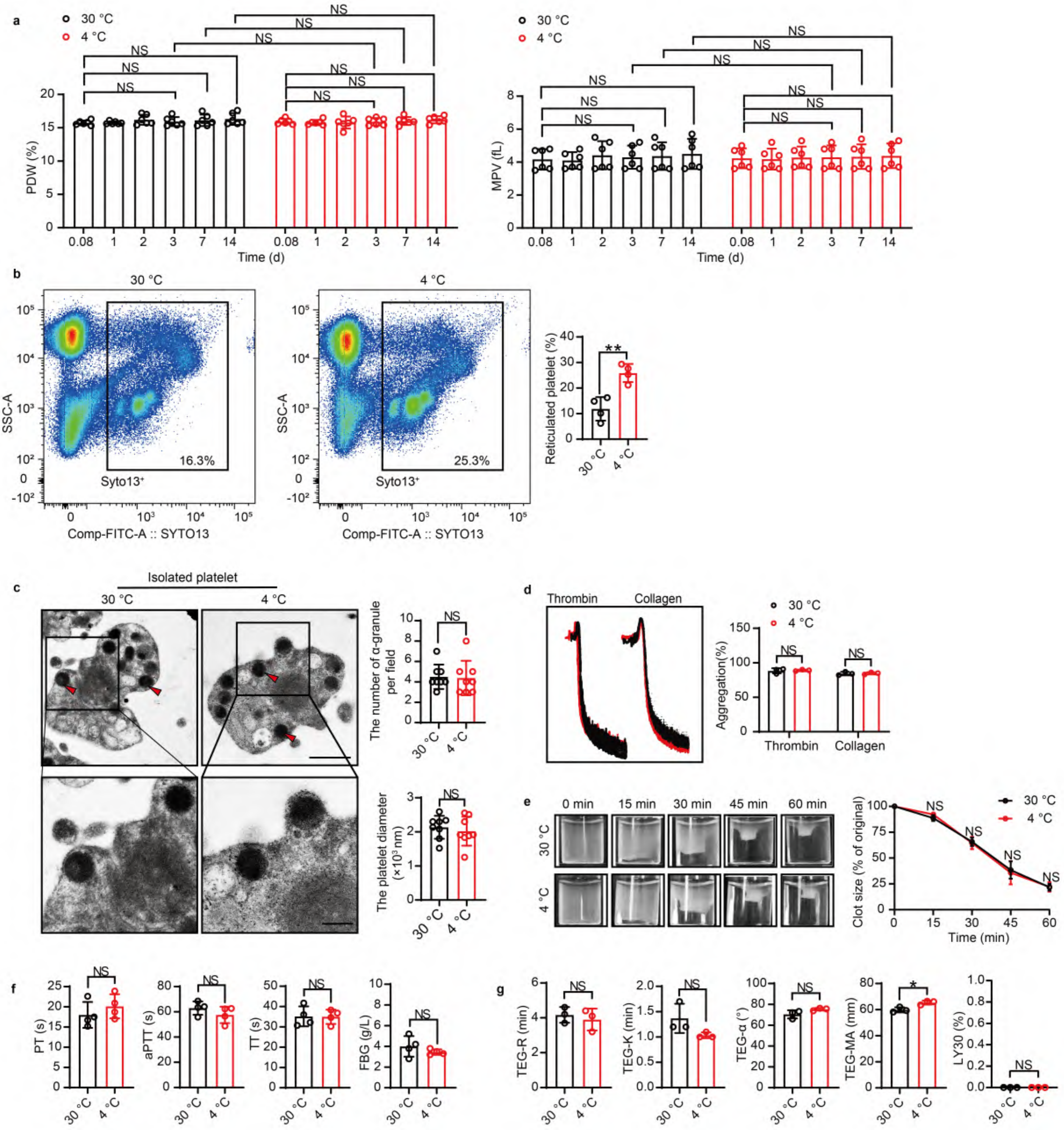
a
30 °C
4 °C
NS
PDW (%)
Time (d)
0.08
1
2
3
7
14
MPV (fL)
b
30 °C
4 °C
SSC-A
Comp-FITC-A :: SYTO13
Syto13⁺
16.3%
25.3%
Reticulated platelet (%)
**
c
Isolated platelet
The number of α-granule per field
The platelet diameter (×10³ nm)
d
Thrombin
Collagen
Aggregation(%)
e
0 min
15 min
30 min
45 min
60 min
Clot size (% of original)
Time (min)
f
PT (s)
aPTT (s)
TT (s)
FBG (g/L)
g
TEG-R (min)
TEG-K (min)
TEG-α (°)
TEG-MA (mm)
LY30 (%)
*

Fig. S1 Cold exposure insignificantly affects platelet function

a PDW and MPV analysis in PB from mice under 30°C and 4°C at various timepoints (*n* = 6 mice per group). **b** Representative flow cytometry plots of SYTO13$^+$ platelets in peripheral blood from 30°C- and 4°C-exposed mice on day 3. Quantification of SYTO13$^+$ immature/reticulated platelets (*n* = 4 mice per group). **c** Representative transmission electron microscopy micrographs of platelets from mice under 30°C and 4°C. Red arrowhead: α-granule. Scale bar in upper panel: 1 μm. Scale bar in lower panel: 300 nm. Quantifications of α-granule number and platelet diameter (*n* = 8 random fields per group). **d** Representative aggregometry tracings of platelet-rich plasma from mice under 30°C and 4°C in response to thrombin or collagen (*n* = 3 mice per group). **e** Representative clot retraction micrographs of platelet-rich plasma from mice under 30°C and 4°C in response to thrombin. Quantifications of the clot retraction analysis (*n* = 3 mice per group). **f** Prothrombin time (PT), thrombin time (TT), activated partial thromboplastin time (aPTT), and fibrinogen concentration (FBG) analysis on blood from mice under 30°C and 4°C (*n* = 4 mice per group). **g** Thromboelastographic (TEG) analysis including reaction time (R), K time (K), α-angle (Angle), and maximum amplitude (MA) in peripheral blood from mice under 30°C and 4°C (*n* = 3 mice per group) *p<0.05; **p<0.01; ***p<0.001. NS = not significant. Data presented as mean ± SD.
