# Supplementary material for: Cold exposure aggravates vein occlusion through non-shivering thermogenesis-induced thrombocytopoiesis
Source: Cell Res. 2026 Jul 30;36(9):698–717. doi: 10.1038/s41422-026-01280-2 (PMC13503729; doi:10.1038/s41422-026-01280-2)
Supplement: Supplementary file 7 [file 41422_2026_1280_MOESM2_ESM.pdf]

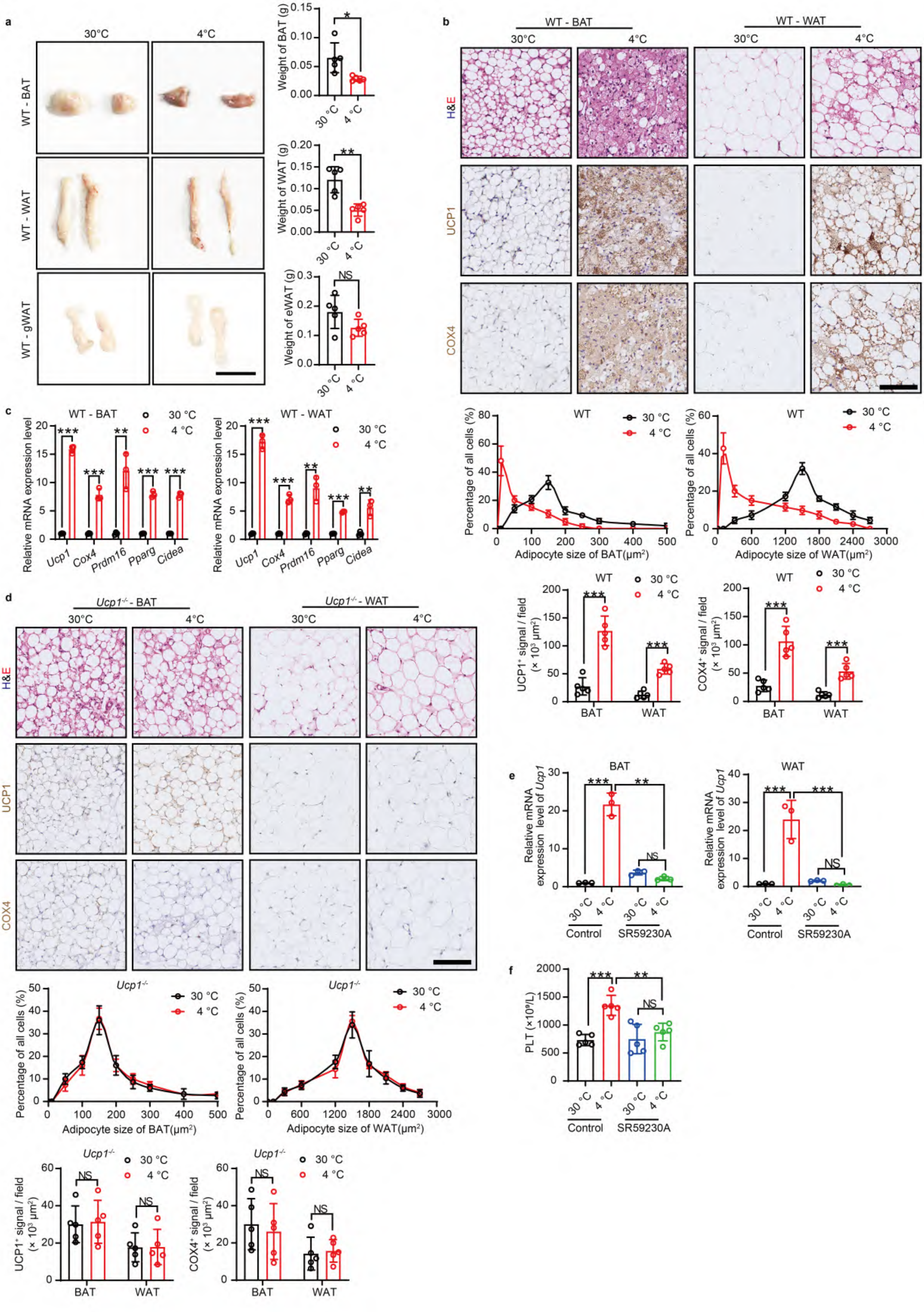

a
30°C
4°C
WT - BAT
WT - WAT
WT - gWAT
Weight of BAT (g)
Weight of WAT (g)
Weight of eWAT (g)
NS
b
WT - BAT
WT - WAT
30°C
4°C
H&E
UCP1
COX4
Percentage of all cells (%)
Adipocyte size of BAT(μm²)
Adipocyte size of WAT(μm²)
WT
30 °C
4 °C
UCP1⁺ signal / field (× 10³ μm²)
COX4⁺ signal / field (× 10³ μm²)
BAT
WAT
c
WT - BAT
WT - WAT
Relative mRNA expression level
Ucp1
Cox4
Prdm16
Pparg
Cidea
d
Ucp1-/- - BAT
Ucp1-/- - WAT
Ucp1-/-
e
Relative mRNA expression level of Ucp1
Control
SR59230A
f
PLT (×10⁹/L)

Fig. S2 Cold exposure promotes adipose tissue browning via UCP1

a Gross pictures and weight quantifications of BAT, WAT (inguinal WAT), and gWAT (*n* = 5 samples per group). Scale bar: 1 cm. **b** Histological and immunohistochemical staining of BAT and WAT with UCP1 and COX4, followed by counterstaining with DAPI (blue) in mice under 30°C and 4°C. Quantifications of adipocyte size, $UCP1^+$ signals, and $COX4^+$ signals (*n* = 5 random fields per group). Scale bar: 100 µm. **c** QPCR quantifications of browning markers in BAT and WAT in mice under 30°C and 4°C (*n* = 3 samples per group). **d** Histological and immunohistochemical staining of BAT and WAT with UCP1 and COX4, followed by counterstaining with DAPI (blue) in $Ucp1^{-/-}$ mice under 30°C and 4°C. Quantifications of adipocyte size, $UCP1^+$ signals, and $COX4^+$ signals (*n* = 5 random fields per group). Scale bar: 100 µm. **e** qPCR quantification of *Ucp1* in BAT and WAT from 30°C- and 4°C-exposed mice treated with or without β3-adrenergic receptor antagonist (*n* = 3 mice per group). **f** Platelet counts in 30°C- and 4°C-exposed mice treated with or without β3-adrenergic receptor antagonist (*n* = 5 mice per group). *p<0.05; **p<0.01; ***p<0.001. NS = not significant. Data presented as mean ± SD.
