# Supplementary material for: Cold exposure aggravates vein occlusion through non-shivering thermogenesis-induced thrombocytopoiesis
Source: Cell Res. 2026 Jul 30;36(9):698–717. doi: 10.1038/s41422-026-01280-2 (PMC13503729; doi:10.1038/s41422-026-01280-2)
Supplement: Supplementary file 8 [file 41422_2026_1280_MOESM3_ESM.pdf]

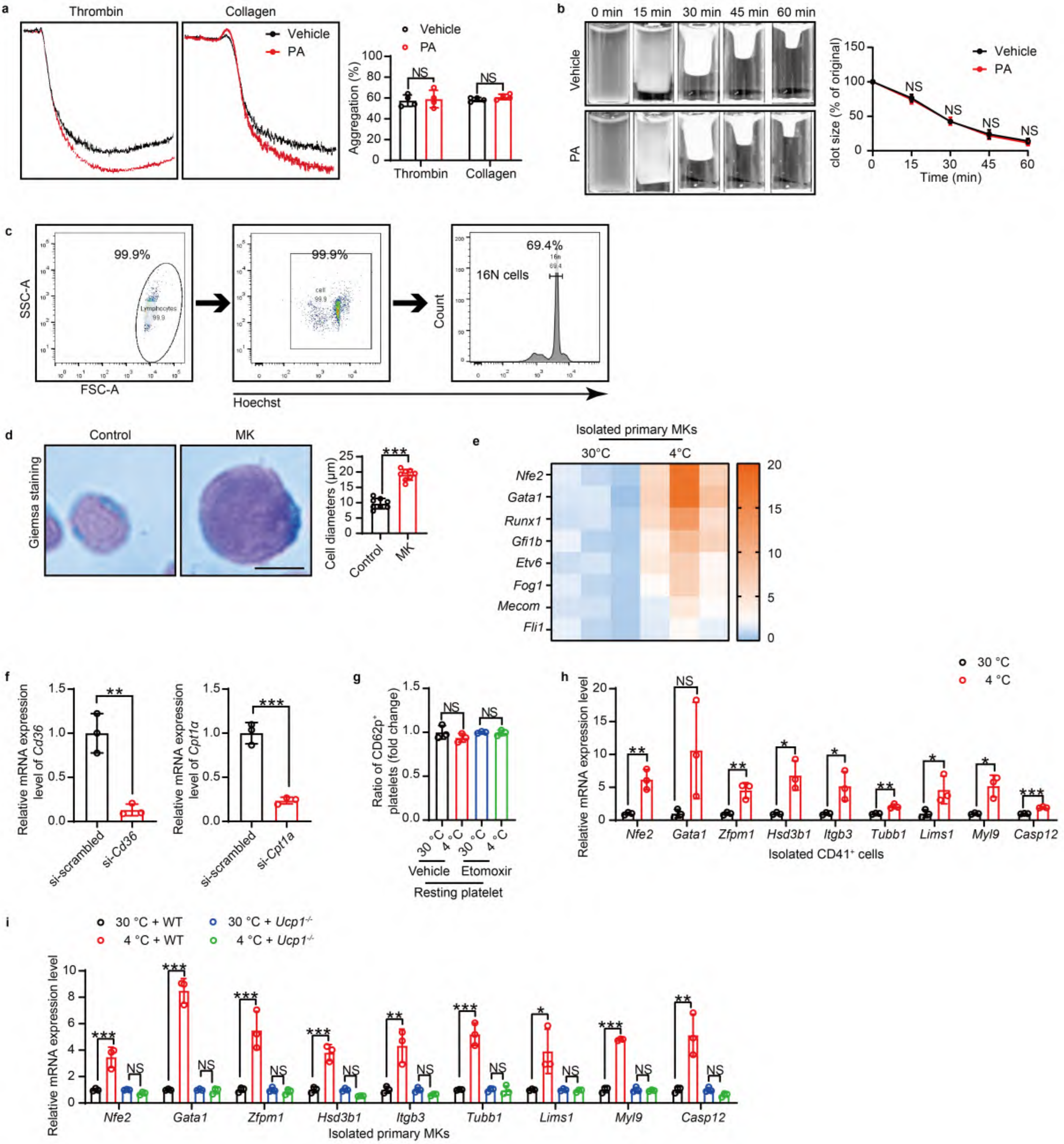

a
Thrombin
Collagen
Vehicle
PA
Aggregation (%)
NS
Thrombin
Collagen
b
0 min
15 min
30 min
45 min
60 min
Vehicle
PA
clot size (% of original)
Time (min)
NS
c
99.9%
99.9%
69.4%
16N cells
SSC-A
FSC-A
Count
Hoechst
d
Control
MK
Giemsa staining
Cell diameters (μm)
***
e
Isolated primary MKs
30°C
4°C
Nfe2
Gata1
Runx1
Gfi1b
Etv6
Fog1
Mecom
Fli1
f
Relative mRNA expression level of Cd36
Relative mRNA expression level of Cpt1α
si-scrambled
si-Cd36
si-Cpt1a
g
Ratio of CD62p+ platelets (fold change)
Vehicle
Etomoxir
Resting platelet
h
30 °C
4 °C
Relative mRNA expression level
Nfe2
Gata1
Zfpm1
Hsd3b1
Itgb3
Tubb1
Lims1
Myl9
Casp12
Isolated CD41+ cells
i
30 °C + WT
4 °C + WT
30 °C + Ucp1-/-
4 °C + Ucp1-/-
Relative mRNA expression level
Isolated primary MKs

Fig. S3 NST-induced thrombocytopoiesis

a Representative aggregometry tracings of platelet-rich plasma from vehicle- or PA-treated mice in response to thrombin or collagen (*n* = 4 mice per group). **b** Representative clot retraction micrographs of platelet-rich plasma from vehicle- or PA-treated mice in response to thrombin. Quantifications of the clot retraction analysis (*n* = 4 mice per group). **c** Hoechst staining analysis of 16N polyploidy cells in isolated primary MKs by flow cytometry. **d** Representative Giemsa staining of isolated primary MKs and non-MKs. Scale bar: 10 μm. Quantifications of cell diameter of isolated primary MKs and non-MKs (*n* = 8 random cells per group). **e** Transcriptomic MK lineage-related TF profiling from isolated primary MKs from mice under 30°C and 4°C (*n* = 3 mice per group). Heatmap of selected TFs. **f** QPCR quantifications of *Cd36* and *Cpt1a* in si-scrambled, si-*Cd36*, or si-*Cpt1α*-transfected primary MKs (*n* = 3 samples per group). **g** Quantification of flow cytometry analysis of $CD62p^+$ resting platelets from vehicle- or etomoxir-treated mice under 30°C and 4°C (*n* = 3 mice per group). **h** Thrombocytopoiesis-associated gene expression of FACS sorted $CD41^+$ cells from mice under 30°C and 4°C (*n* = 3 samples per group). **i** Thrombocytopoiesis-associated gene expression of isolated primary MKs from $Ucp1^{-/-}$ mice under 30°C and 4°C (*n* = 3 samples per group). *p<0.05; **p<0.01; ***p<0.001. NS = not significant. Data presented as mean ± SD.
