# Supplementary material for: Cold exposure aggravates vein occlusion through non-shivering thermogenesis-induced thrombocytopoiesis
Source: Cell Res. 2026 Jul 30;36(9):698–717. doi: 10.1038/s41422-026-01280-2 (PMC13503729; doi:10.1038/s41422-026-01280-2)
Supplement: Supplementary file 9 [file 41422_2026_1280_MOESM4_ESM.pdf]

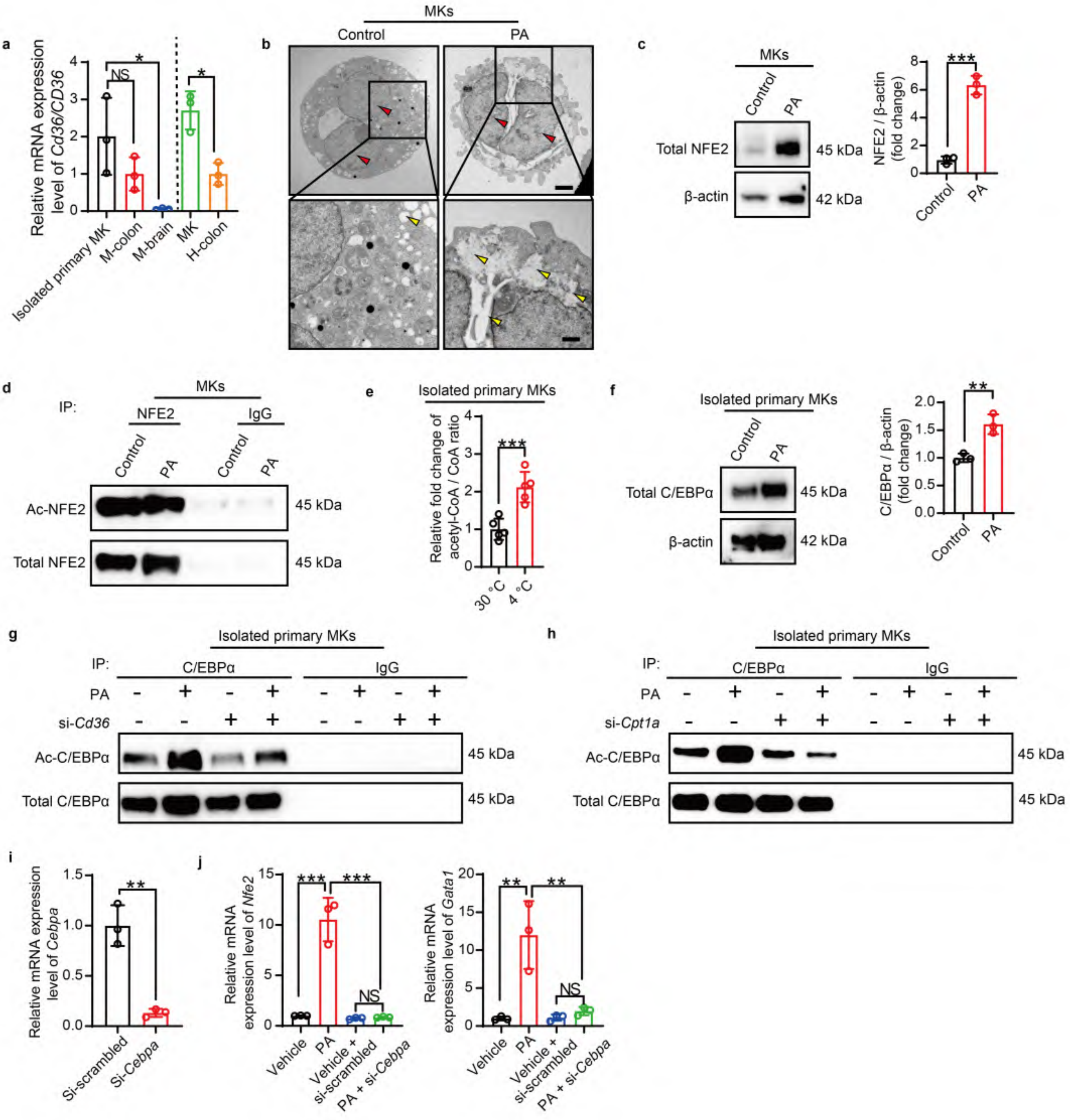
a
Relative mRNA expression level of Cd36/CD36
NS
*
*
Isolated primary MK
M-colon
M-brain
MK
H-colon
b
MKs
Control
PA
c
MKs
Control
PA
Total NFE2
β-actin
45 kDa
42 kDa
NFE2 / β-actin (fold change)
***
d
IP:
MKs
NFE2
IgG
Control
PA
Ac-NFE2
Total NFE2
45 kDa
e
Isolated primary MKs
Relative fold change of acetyl-CoA / CoA ratio
***
30 °C
4 °C
f
Isolated primary MKs
Control
PA
Total C/EBPα
β-actin
45 kDa
42 kDa
C/EBPα / β-actin (fold change)
**
g
Isolated primary MKs
IP:
C/EBPα
IgG
PA
si-Cd36
Ac-C/EBPα
Total C/EBPα
45 kDa
h
Isolated primary MKs
IP:
C/EBPα
IgG
PA
si-Cpt1a
Ac-C/EBPα
Total C/EBPα
45 kDa
i
Relative mRNA expression level of Cebpa
**
Si-scrambled
Si-Cebpa
j
Relative mRNA expression level of Nfe2
***
***
NS
Vehicle
PA
Vehicle + si-scrambled
PA + si-Cebpa
Relative mRNA expression level of Gata1
**
**
NS

Fig. S4 C/EBPα acetylation in isolated primary MKs

a QPCR analysis of *Cd36* in mouse primary MKs and *CD36* in human MK cell line (*n* = 3 samples per group). Mouse and human colon tissues were used as positive controls. **b** Representative transmission electron microscopy micrographs of MKs from vehicle- or PA-treated MKs. Red arrowhead: nucleus. Yellow arrowhead: pre-DMS. Scale bar in upper panel: 1 μm. Scale bar in lower panel: 500 nm. **c** Protein expression levels of NF-E2 in DMSO control- or PA-treated MKs (*n* = 3 samples per group). β-actin marked the loading level in each line. **d** IP of NF-E2 in control- or PA-treated MKs. Acetyl-NF-E2 was detected. Non-immune IgG was used as a negative control. **e** ELISA detection of intracellular acetyl-CoA/CoA ratio in isolated BM MKs from 30°C- and 4°C-treated mice (*n* = 5 samples per group). **f** Protein expression levels of C/EBPα in control- or PA-treated primary BM MKs (*n* = 3 samples per group). β-actin marked the loading level in each line. **g, h** IP of C/EBPα in control- or PA-treated primary BM MKs with or without si-*Cd36* (**g**) or si-*Cpt1a* administration (**h**). Acetyl-C/EBPα was detected. Non-immune IgG was used as a negative control. **i** QPCR quantifications of *Cebpa* in si-scrambled or si-*Cebpa*-transfected primary MKs (*n* = 3 samples per group). **j** QPCR quantifications of *Nfe2* and *Gata1* in si-scrambled or si-*Cebpa*-transfected primary MKs under vehicle or PA administration (*n* = 3 samples per group). *p<0.05; **p<0.01; ***p<0.001. NS = not significant. Data presented as mean ± SD.
