# Supplementary material for: Cold exposure aggravates vein occlusion through non-shivering thermogenesis-induced thrombocytopoiesis
Source: Cell Res. 2026 Jul 30;36(9):698–717. doi: 10.1038/s41422-026-01280-2 (PMC13503729; doi:10.1038/s41422-026-01280-2)
Supplement: Supplementary file 10 [file 41422_2026_1280_MOESM5_ESM.pdf]

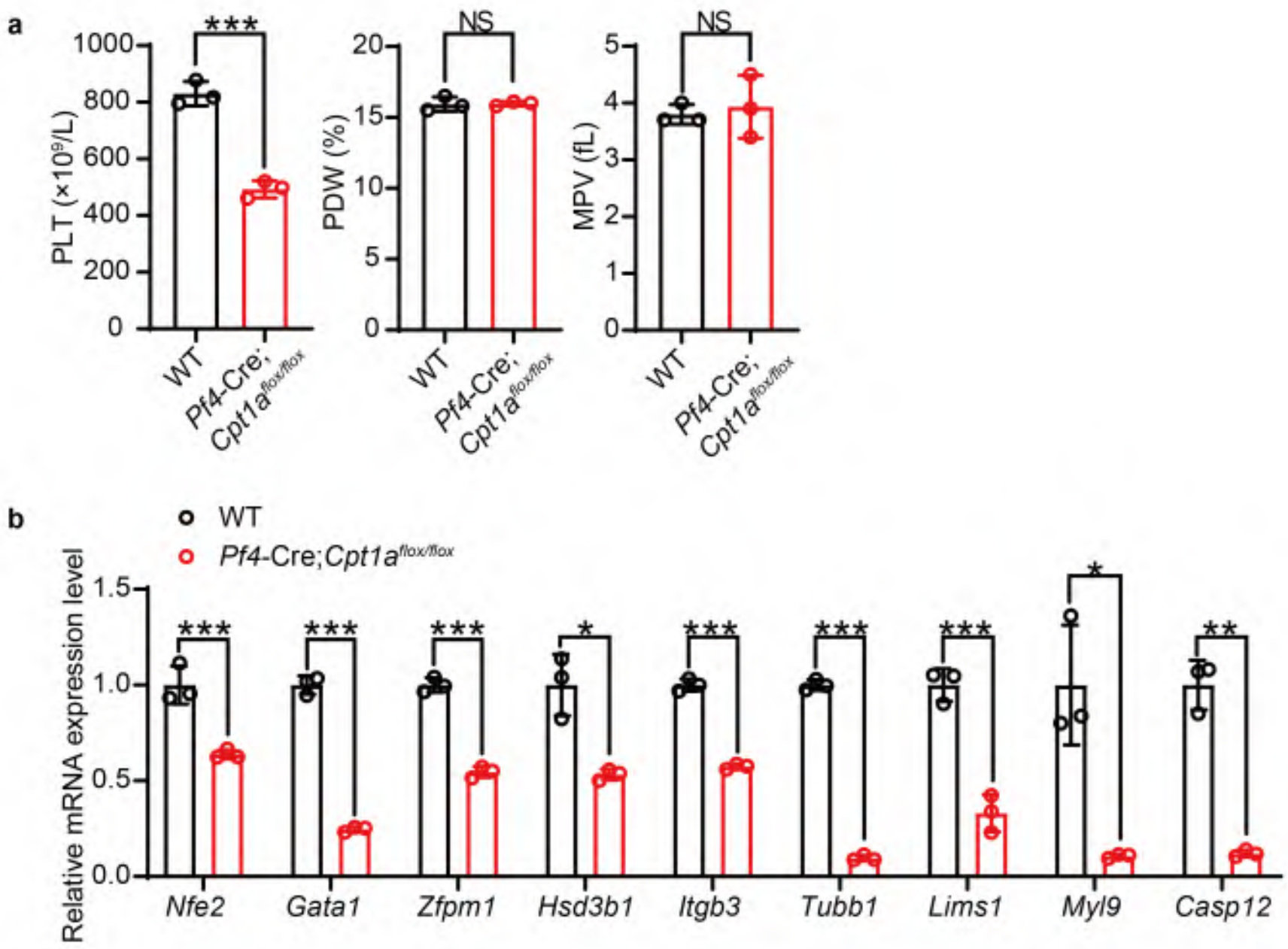
a
PLT (×10⁹/L)
PDW (%)
MPV (fL)
***
NS
NS
WT
Pf4-Cre;Cpt1a flox/flox
b
WT
Pf4-Cre;Cpt1a flox/flox
Relative mRNA expression level
Nfe2
Gata1
Zfpm1
Hsd3b1
Itgb3
Tubb1
Lims1
Myl9
Casp12

Fig. S5 Genetic depletion of *Cpt1a* in MKs reduces the platelet count in mice

a PLT, PCT, PDW, and MPV analysis in PB from wt mice and *Pf4*-Cre *Cpt1a*$^{flox/flox}$ mice (*n* = 3 mice per group). **b** Thrombocytopoiesis-associated gene expression in isolated BM MKs from wt mice and *Pf4*-Cre *Cpt1a*$^{flox/flox}$ mice (*n* = 3 samples per group). *p<0.05; **p<0.01; ***p<0.001. NS = not significant. Data presented as mean ± SD.
