# Supplementary material for: Cold exposure aggravates vein occlusion through non-shivering thermogenesis-induced thrombocytopoiesis
Source: Cell Res. 2026 Jul 30;36(9):698–717. doi: 10.1038/s41422-026-01280-2 (PMC13503729; doi:10.1038/s41422-026-01280-2)
Supplement: Supplementary file 11 [file 41422_2026_1280_MOESM6_ESM.pdf]

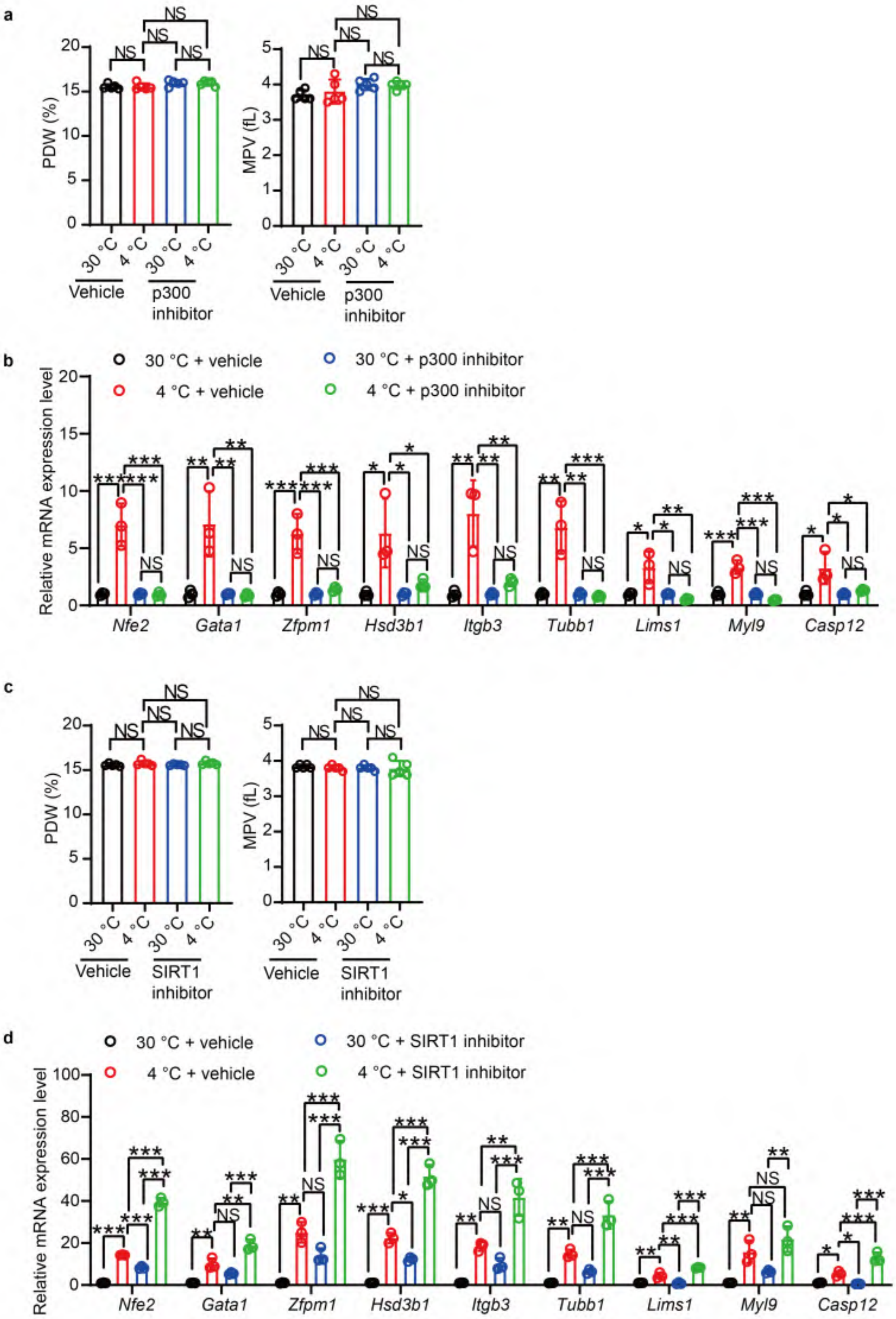
a
PDW (%)
MPV (fL)
30 °C
4 °C
Vehicle
p300 inhibitor
NS
b
30 °C + vehicle
4 °C + vehicle
30 °C + p300 inhibitor
4 °C + p300 inhibitor
Relative mRNA expression level
Nfe2
Gata1
Zfpm1
Hsd3b1
Itgb3
Tubb1
Lims1
Myl9
Casp12
c
PDW (%)
MPV (fL)
Vehicle
SIRT1 inhibitor
d
30 °C + vehicle
4 °C + vehicle
30 °C + SIRT1 inhibitor
4 °C + SIRT1 inhibitor
Relative mRNA expression level

Fig. S6 Targeting p300 or SIRT1 for regulating thrombocytopoiesis-associated gene expression

a Platelet parameters in vehicle- or p300 inhibitor-treated mice under 30°C and 4°C (*n* = 5 mice per group). **b** Thrombocytopoiesis-associated gene expression in isolated MKs from vehicle- or p300 inhibitor-treated mice under 30°C and 4°C (*n* = 3 samples per group). **c** Platelet parameters from vehicle- or SIRT1 inhibitor-treated mice under 30°C and 4°C (*n* = 5 mice per group). **d** Thrombocytopoiesis-associated gene expression in isolated MKs from vehicle- or SIRT1 inhibitor-treated mice under 30°C and 4°C (*n* = 3 samples per group). *p<0.05; **p<0.01; ***p<0.001. NS = not significant. Data presented as mean ± SD.
