# Supplementary material for: Cold exposure aggravates vein occlusion through non-shivering thermogenesis-induced thrombocytopoiesis
Source: Cell Res. 2026 Jul 30;36(9):698–717. doi: 10.1038/s41422-026-01280-2 (PMC13503729; doi:10.1038/s41422-026-01280-2)
Supplement: Supplementary file 12 [file 41422_2026_1280_MOESM7_ESM.pdf]

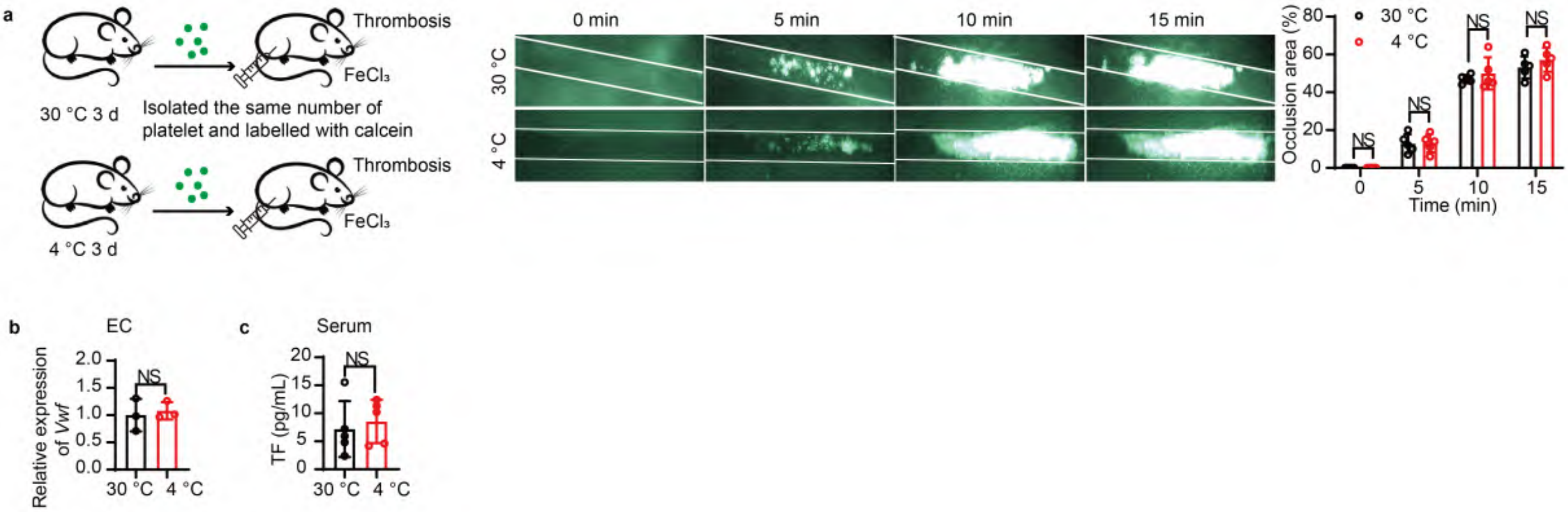

a
Thrombosis
FeCl3
30 °C 3 d
Isolated the same number of
platelet and labelled with calcein
Thrombosis
FeCl3
4 °C 3 d
0 min
5 min
10 min
15 min
30 °C
4 °C
Occlusion area (%)
30 °C
4 °C
NS
Time (min)
b
EC
Relative expression of Vwf
NS
30 °C
4 °C
c
Serum
TF (pg/mL)
NS
30 °C
4 °C

**Fig. S7 Cold exposure insignificantly alters the thrombi-forming ability per platelet**

**a** Schematic figure of thrombosis formation model receiving platelet transfusion of same number of labeled platelet from 30°C- and 4°C-exposed mice. Representative thrombi micrographs upon $FeCl_3$-induced endothelium injury with platelets from mice under 30°C and 4°C. Quantifications of $FeCl_3$-induced occlusion at various timepoints (*n* = 5 samples per group). **b** QPCR quantifications of *Vwf* in isolated endothelial cells from IVC in 30°C- and 4°C-exposed mice (*n* = 3 samples per group). **c** Serum tissue factor levels in 30°C- and 4°C-exposed mice (*n* = 5 samples per group). *p<0.05; **p<0.01; ***p<0.001. NS = not significant. Data presented as mean ± SD.
