# Supplementary material for: Cold exposure aggravates vein occlusion through non-shivering thermogenesis-induced thrombocytopoiesis
Source: Cell Res. 2026 Jul 30;36(9):698–717. doi: 10.1038/s41422-026-01280-2 (PMC13503729; doi:10.1038/s41422-026-01280-2)
Supplement: Supplementary file 13 [file 41422_2026_1280_MOESM8_ESM.pdf]

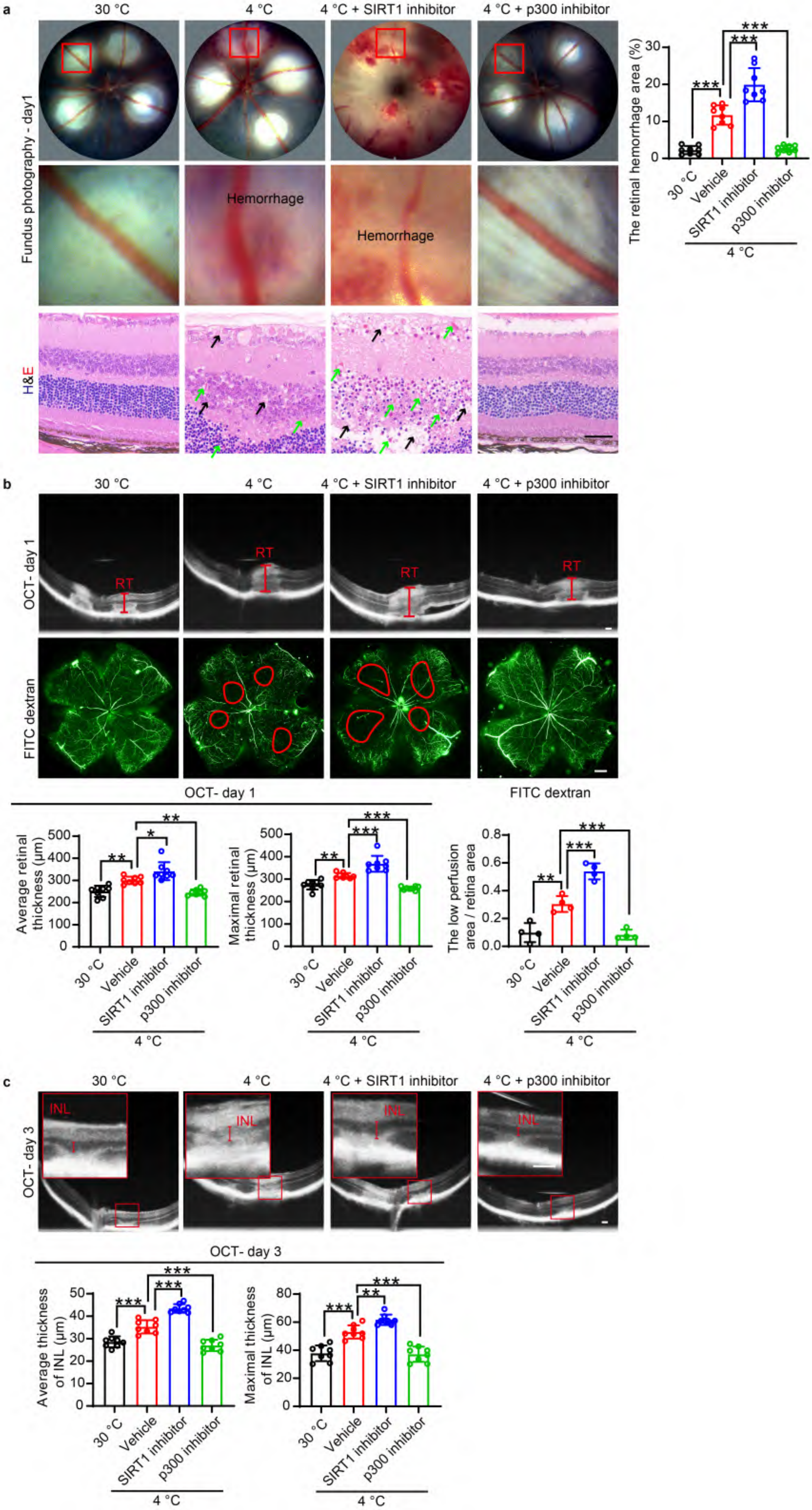
a
30 °C
4 °C
4 °C + SIRT1 inhibitor
4 °C + p300 inhibitor
Fundus photography - day1
Hemorrhage
Hemorrhage
H&E
The retinal hemorrhage area (%)
30 °C
Vehicle
SIRT1 inhibitor
p300 inhibitor
4 °C
b
30 °C
4 °C
4 °C + SIRT1 inhibitor
4 °C + p300 inhibitor
OCT- day 1
RT
FITC dextran
OCT- day 1
FITC dextran
Average retinal thickness (μm)
Maximal retinal thickness (μm)
The low perfusion area / retina area
30 °C
Vehicle
SIRT1 inhibitor
p300 inhibitor
4 °C
c
30 °C
4 °C
4 °C + SIRT1 inhibitor
4 °C + p300 inhibitor
OCT- day 3
INL
OCT- day 3
Average thickness of INL (μm)
Maximal thickness of INL (μm)
30 °C
Vehicle
SIRT1 inhibitor
p300 inhibitor
4 °C

Fig. S8 Targeting acetylation for modulating cold-aggravated RVO

a Representative fundus photography and histological analysis of 30°C- and 4°C-exposed mice with or without SIRT1 inhibitor and p300 inhibitor. Black arrow: cystoid edema. Green arrow: hemorrhage area. Scale bar: 50 µm. Quantification of retinal hemorrhage area in various groups (*n* = 8 eyes per group). **b** 24 h after laser irradiation, representative OCT image at the laser-irradiated point and capillary perfusion detection by retinal angiography in 30°C- and 4°C-exposed mice with or without SIRT1 inhibitor and p300 inhibitor. RT: Retinal thickness. Dashed lines marked non-perfused capillary area. Scale bar in upper panel: 100 µm. Scale bar in lower panel: 500 µm. Quantification of retinal thickness (*n* = 8 eyes per group) and capillary perfusion in various groups (*n* = 4 eyes per group). **c** 72 h after laser irradiation, representative OCT image at the laser-irradiated point in 30°C- and 4°C-exposed mice with or without SIRT1 inhibitor and p300 inhibitor. INL: Inter nuclear layer thickness. Scale bar: 100 µm. Quantification of INL thickness in various groups (*n* = 8 eyes per group). *p<0.05; **p<0.01; ***p<0.001. NS = not significant. Data presented as mean ± SD.
