# Supplementary material for: Cold exposure aggravates vein occlusion through non-shivering thermogenesis-induced thrombocytopoiesis
Source: Cell Res. 2026 Jul 30;36(9):698–717. doi: 10.1038/s41422-026-01280-2 (PMC13503729; doi:10.1038/s41422-026-01280-2)
Supplement: Supplementary file 14 [file 41422_2026_1280_MOESM9_ESM.pdf]

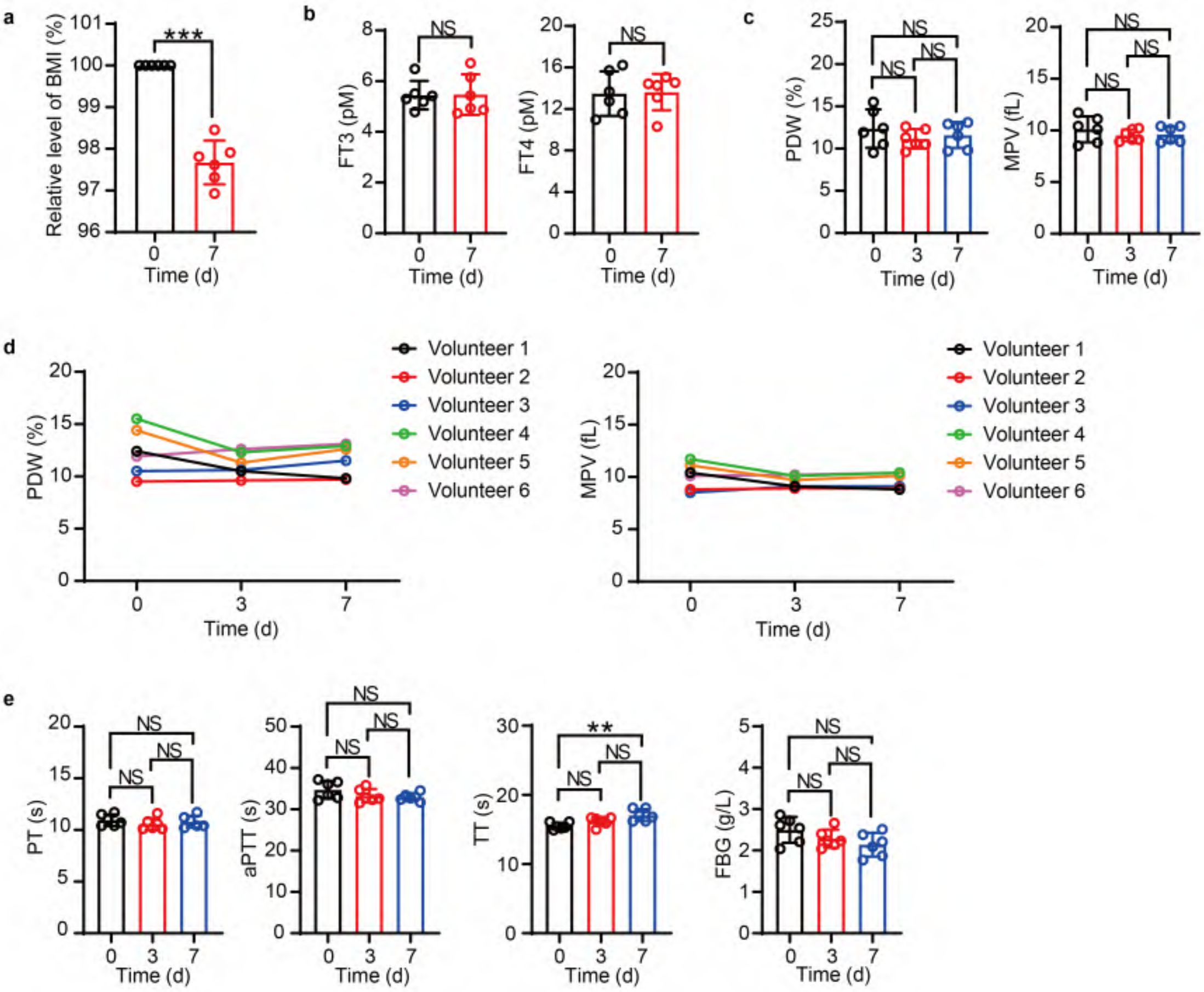
a
Relative level of BMI (%)
***
Time (d)
b
FT3 (pM)
FT4 (pM)
NS
Time (d)
c
PDW (%)
MPV (fL)
NS
Time (d)
d
PDW (%)
MPV (fL)
Volunteer 1
Volunteer 2
Volunteer 3
Volunteer 4
Volunteer 5
Volunteer 6
Time (d)
e
PT (s)
aPTT (s)
TT (s)
FBG (g/L)
NS
**
Time (d)

Fig. S9 Mild cold exposure reduces BMI in humans

a Quantification of BMI fold change before and after 7-day cold exposure (*n* = 6 subjects per group). **b** Quantification of circulating free T3 and free T4 levels before and after 7-day cold exposure (*n* = 6 subjects per group). **c, d** Fold change and individual analysis of PDW and MPV analysis in PB on day 0, 3, 7 from volunteers under cold exposure (*n* = 6 subjects per group). **e** Prothrombin time (PT), thrombin time (TT), activated partial thromboplastin time (aPTT), and fibrinogen concentration (FBG) analysis on day 0, 3, 7 from volunteers under cold exposure (*n* = 6 subjects per group). *p<0.05; **p<0.01; ***p<0.001. NS = not significant. Data presented as mean ± SD.
